# Temporal dynamics of incomplete lineage sorting, introgression and divergence in eucalypts distributed across an expansive and ancient landscape

**DOI:** 10.1093/aob/mcag242

**Published:** 2026-08-05

**Authors:** Harvey K Orel, Rachael M Fowler, Todd G B McLay, David J Cantrill, Donald C Franklin, Daniel J Murphy, Michael J Bayly

**Affiliations:** School of BioSciences, The University of Melbourne, Parkville, Victoria 3010, Australia; School of BioSciences, The University of Melbourne, Parkville, Victoria 3010, Australia; Royal Botanic Gardens Victoria, South Yarra, Victoria 3141, Australia; School of BioSciences, The University of Melbourne, Parkville, Victoria 3010, Australia; Royal Botanic Gardens Victoria, South Yarra, Victoria 3141, Australia; National Biodiversity DNA Library, National Research Collections Australia, CSIRO, Parkville, Victoria 3052, Australia; Royal Botanic Gardens Victoria, South Yarra, Victoria 3141, Australia; Research Institute for the Environment & Livelihoods, Charles Darwin University, Darwin, Northern Territory 0909, Australia; Royal Botanic Gardens Victoria, South Yarra, Victoria 3141, Australia; School of BioSciences, The University of Melbourne, Parkville, Victoria 3010, Australia

**Keywords:** *Angophora*, Australia, *Blakella*, *Corymbia*, evolution, hybridization, phylogenomic discordance, sex-biased dispersal, syngameon

## Abstract

**Background and Aims:**

The eucalypt clade composed of *Angophora*, *Blakella* and *Corymbia* (the ‘bloodwood eucalypts’) includes >110 species and dominates the vast savannahs, open forests and woodlands of northern Australia. Relationships within this clade have been difficult to decipher despite significant previous investigation.

**Methods:**

Using target-capture data from a eucalypt-specific bait set (568 nuclear genes) and whole plastome sequences, we conducted a phylogenomic study of *Angophora*, *Blakella* and *Corymbia* (sampling 97 species with 168 accessions) using tree, network, divergence dating and coalescent simulation analyses to investigate evolutionary relationships and patterns of phylogenomic discordance.

**Key Results:**

Phylogenetic relationships in *Angophora*, *Blakella* and *Corymbia* are affected by extensive phylogenomic discordance, attributed to persistently high levels of incomplete lineage sorting through time and frequent introgression concentrated at particular branches. Branches affected by introgression were consistently co-affected by incomplete lineage sorting, and nuclear genes with higher recombination rates more frequently supported discordant topologies affected by incomplete lineage sorting. Additionally, a mixture of incomplete lineage sorting, plastid capture and sex-biased dispersal are likely to be responsible for substantial cytonuclear discordance. Based on molecular divergence dating analyses, diversification of lineages within genera was estimated to have commenced during the late Oligocene.

**Conclusions:**

We provide a robust framework phylogeny for *Angophora*, *Blakella* and *Corymbia* and further support for the position of *Blakella* as sister to *Angophora* and *Corymbia*. We attribute the difficulty in resolving this trichotomy to incomplete lineage sorting during divergence, with subsequent hybridization between *Blakella* and *Corymbia*. Patterns of high discordance, incomplete lineage sorting and introgression across the phylogeny indicate that evolution at the genomic level does not follow a single, bifurcating species tree. Ultimately, our results demonstrate the intrinsic entwinement of diversification and discordance-inducing processes in the evolutionary history of eucalypts and highlight the group as an example of a phylogenomic mosaic.

## INTRODUCTION

Widespread adoption of genomic-scale data has seen a shift in the way that discordance is considered in phylogenomic studies. Although the challenge presented by phylogenetic discordance is not new (Rieseberg *et al.*, 1990; Doyle, 1992; Maddison, 1997), greater emphasis has recently been placed on trying to understand sources of discordance and how they relate to aspects of evolutionary and biological history (Haber, 2019; Smith *et al.*, 2020; Morales-Briones *et al.*, 2021; Rose *et al.*, 2021; Joyce *et al.*, 2025). The term ‘phylogenetic discordance’ is used to describe disagreements between data used for reconstructing phylogenies (Larson *et al.*, 2026). For genomic data, disagreements stemming from biological processes can occur across and within various genomic compartments. In plants, cytonuclear discordance between phylogenies derived from organellar (i.e. plastid and mitochondrial) and nuclear DNA sequence data may result from introgression and plastid capture following hybridization, incomplete lineage sorting (ILS), horizontal and intracellular gene transfer, or by differential cladogenesis owing to different modes of inheritance and unequal, sex-biased dispersal between pollen and seeds (Birky *et al.*, 1983; Rieseberg *et al.*, 1996; Irwin, 2002; Kuo and Avise, 2005). Within the nuclear genome, discordance between independent loci can arise from introgression following hybridization, ILS, selection, and gene or whole genome duplications and loss (Maddison, 1997; Rannala *et al.*, 2020). Although attributing genomic discordance to a particular source can be difficult, attempting to do so can provide valuable evolutionary insight.

Of these phenomena, two of the most prevalent in plants are ILS and hybridization (Rieseberg *et al.*, 1996; Maddison, 1997; Rieseberg and Carney, 1998; Soltis and Soltis, 2009; Stull *et al.*, 2023). Both can lead to cytonuclear and nuclear–nuclear discordance and, given that they are more likely in closely related lineages with short internal branches, they tend to occur together (Pamilo and Nei, 1988; Mallet, 2005; Maddison and Knowles, 2006). Their effects are biologically and evolutionarily varied; ILS may act as a buffer to fixation by genetic drift via retention of large effective population sizes through speciation (Rivas-González *et al.*, 2023), whereas hybridization can increase genetic diversity, enhance adaptive mechanisms, facilitate acquisition of favourable alleles and enable expansion into new niches (Rieseberg, 1997; Goulet *et al.*, 2017; Buck *et al.*, 2023) or, negatively, result in accumulation of deleterious alleles, genetic assimilation and hybrid sterility (Levin *et al.*, 1996; Rieseberg and Carney, 1998; Johansen-Morris and Latta, 2006; Moran *et al.*, 2021). Hybridization and ILS are prevalent in many hyper-dominant tree genera, including oaks (*Quercus*; Zhou *et al.*, 2022; Liu *et al.*, 2025), pines (*Pinus*; Zhao *et al.*, 2024), poplars (*Populus*; Wang *et al.*, 2020) and willows (*Salix*; Sanderson *et al.*, 2023), and how they relate to the success of such lineages is a key question in evolutionary biology.

In Australia, the eucalypts (Myrtaceae: tribe Eucalypteae) are a landscape-defining plant group in which hybridization and ILS are prominent (Drake, 1980; Griffin *et al.*, 1988; Hill and Johnson, 1995; McKinnon *et al.*, 2004; McLay *et al.*, 2023). These processes have long hindered efforts to resolve evolutionary relationships in the group as bifurcating phylogenies using molecular data (Steane *et al.*, 2002; Parra-O *et al.*, 2006; Gibbs *et al.*, 2009; Fahey *et al.*, 2022).

Within the eucalypts, two lineages host most of the ∼850 total species; one of these is the megadiverse genus *Eucalyptus* (∼750 spp.) and the other, which is the focus of the present study, contains three genera (*sensu*Crisp *et al.*, 2024): *Angophora* (11 spp.) and the ‘bloodwood eucalypts’, *Blakella* (∼38 spp.) and *Corymbia* (∼62 spp.). Although some species of this second lineage occur in mesic regions of south-eastern and south-western Australia, the bloodwood eucalypts are most prominent throughout the savannahs, open forests and woodlands of northern Australia (Fig. 1). They are particularly diverse in the vast tropical savannahs of the region that occupy almost one-quarter of the Australian continent (∼1.9 million km^2^) and are characterized by monsoonal summer rains and dry winters with frequent wildfires (Fox *et al.*, 2001). In these habitats, *Blakella* and *Corymbia* often exhibit higher local species richness than *Eucalyptus* (Franklin and Preece, 2014), and some species are dominant and widespread across thousands of kilometres.

**Fig. 1.**
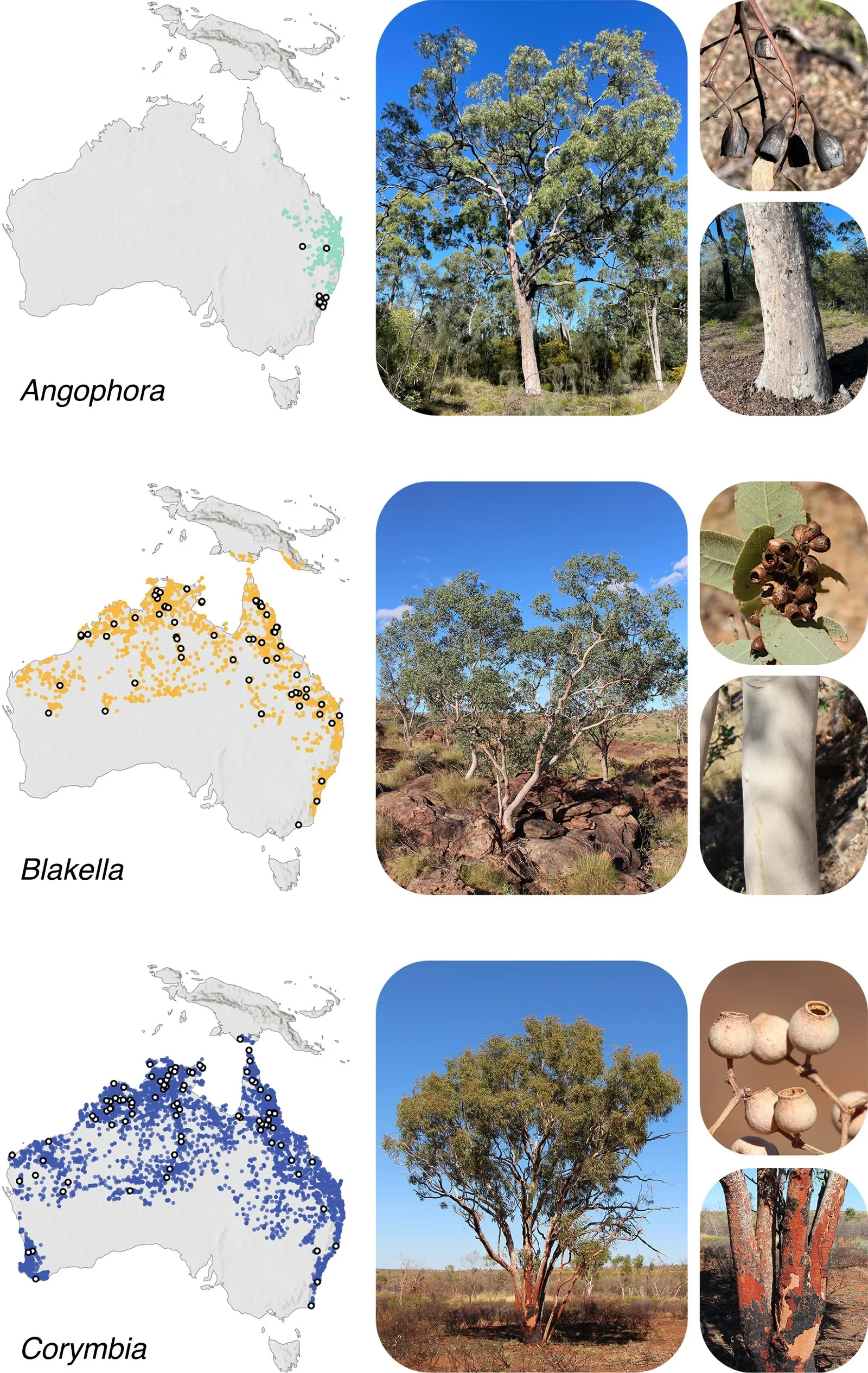
Distribution maps of *Angophora*, *Blakella* and *Corymbia* based on cleaned herbarium records from AVH (2025), with collection localities of samples used in this study for each genus (larger white dots) and images showing exemplar species. Photographs: *A. leiocarpa* (©D. Nicolle; https://www.inaturalist.org/observations/288333578); *B. aspera* (fruits: ©M. Fagg, Australian National Botanic Gardens; else: M. Bayly); and *C. dichromophloia* (M. Bayly).

Phylogenetic studies have consistently resolved *Angophora*, *Blakella* and *Corymbia* together in a well-supported clade (with *Blakella* previously included in *Corymbia* as a subgenus), but relationships between the three genera have been equivocal. Some studies have placed *Angophora* as sister to *Corymbia* and *Blakella* (Udovicic *et al.*, 1995; Ochieng *et al.*, 2007; Parra-O *et al.*, 2009), some have placed *Corymbia* as sister to *Angophora* and *Blakella* (Udovicic and Ladiges, 2000; Steane *et al.*, 2002), and others have placed *Blakella* as sister to *Angophora* and *Corymbia* (Bayly *et al.*, 2008; Thornhill *et al.*, 2019; Crisp *et al.*, 2024). These studies have also shown that taxonomic concepts generally agree with nuclear DNA (nDNA) phylogenies and that there is considerable discordance between nDNA and plastid DNA (ptDNA) phylogenies (Schuster *et al.*, 2018). Previously, nDNA phylogenies of the clade were limited to Sanger sequencing-derived markers and lacked the resolution to clarify the relationships of the genera conclusively (Parra-O *et al.*, 2006, 2009). Recently, a study based on a target capture dataset of 101 nuclear exons found relationships between the genera to be affected by significant genomic discordance, and elevated *Blakella* to the rank of genus without thoroughly investigating this discordance (Crisp *et al.*, 2024). Because of the ecological and economic importance of this group (Williams and Woinarski, 1997) and the fact that outcomes for classification are contentious and topical (see Crisp *et al.*, 2024; Cook *et al.*, 2025; Nicolle *et al.*, 2025), a comprehensive investigation of evolutionary relationships and genomic discordance is warranted.

Here, we conduct a phylogenomic investigation of *Angophora*, *Blakella* and *Corymbia* using a dataset of 568 nuclear genes and whole plastomes from 200 samples, representing 97/∼111 ingroup species. Our aims were to establish a comprehensive framework phylogeny for the group and to examine patterns of nuclear–nuclear and cytonuclear discordance to gain insight into the evolutionary dynamics of processes that induce genomic discordance, such as ILS and hybridization, through time.

## MATERIALS AND METHODS

### Taxon sampling

Leaf material was sourced from herbarium specimens or field-collected specimens dried in silica. Ingroup sampling included 168 samples, with 10 samples from *Angophora* (8/11 species; 2 species with multiple samples), 61 from *Blakella* (34/∼38 described species; 19 species with multiple samples) and 97 from *Corymbia* (55/∼62 described species; 30 species with multiple samples). All current sections of the genera were sampled comprehensively, including *Blakella* sections *Blakella*, *Maculatae* and *Naviculares* (*sensu*Crisp *et al.*, 2024) and *Corymbia* sections *Calophyllae* and *Corymbia* (*sensu*Nicolle, 2022). We included 32 outgroup samples (30 species) from *Eucalyptus*, representing all subgenera (*sensu*Nicolle, 2022). Accession details are listed in Supplementary Data Table S1.

### DNA extraction, library preparation and sequencing

DNA extraction, genomic library preparation and target capture were performed as detailed by McLay *et al.* (2023). Before sequencing, pooled enriched libraries were combined with pooled unenriched libraries in a 3:1 ratio (enriched:unenriched) to increase the proportion of off-target DNA in the final pool. Sequencing was performed at the Australian Genome Research Facility (AGRF, Melbourne, Victoria, Australia) on a NovaSeq 6000 with an S4 300-cycle kit (v.1.5 chemistry, catalogue no.: 20028312).

### Assembly of enriched nuclear loci

Prior to assembly, we used fastp v.0.21.0 (Chen *et al.*, 2018) to remove adapter sequences, poor-quality bases and reads with the following settings: polyG tail trimming (‘-g’), length requirement of 50 bases (‘-l 50’) and a complexity filter (‘-y’) with a 60 % complexity requirement (‘-Y 60’). We then ran an additional filtering step, using Shannon’s entropy (Shannon, 1948) to calculate read diversity and remove low-entropy reads (script available from https://github.com/Royal-Botanic-Gardens-Victoria/DNA_entropy_scripts/blob/main/shannons-filter.py), removing reads with a Shannon index <0.8.

Gene sequences were assembled from the filtered reads using HybPiper v.2.0.1rc (Johnson *et al.*, 2016; Jackson *et al.*, 2023). We used the command ‘hybpiper assemble’ with the target reference file from McLay *et al.* (2023), DIAMOND v.2.0.11 (Buchfink *et al.*, 2021) with default sensitivity for read-to-reference mapping, and SPAdes v.3.15.4 (Bankevich *et al.*, 2012; Prjibelski *et al.*, 2020) with a minimum coverage cut-off value of five for assemblies. The ‘-run_intronerate’ argument was used to recover intron sequences for each sample, and all other settings were left at their defaults. The commands ‘hybpiper stats’, ‘hybpiper retrieve_sequences’ and ‘hybpiper paralog_retriever’ were used to generate assembly statistics and extract sequences.

### Nuclear gene filtering, ortholog inference and consensus sequence generation

#### Gene filtering and ortholog inference

We used a two-step process to filter genes and reconcile orthologs from gene assemblies. Initially, we used the single nucleotide polymorphism assessment step of HybPhaser (Nauheimer *et al.*, 2021) to identify putative paralogs and poorly recovered genes and samples. We then earmarked any genes with <50 % of the reference sequence recovered, <50 % of samples recovered or with a single nucleotide polymorphism proportion >Q3 + 1.5(interquartile range) for removal.

Secondly, we used the ‘paralogs_no_chimeras’ gene sequences output by HybPiper. Genes and samples identified for removal by HybPhaser were removed using Python scripts (available at https://github.com/hkore1/python_scripts). Paralog sequence files were input to ParaGone v.0.0.10 (Jackson *et al.*, 2023) to infer ortholog groups using the tree-based orthology inference algorithms described by Yang and Smith (2014). *Eucalyptus* samples were treated as outgroups, and we ran the default monophyletic outgroups (MO), maximum inclusion (MI) and rooted subtrees (RT) algorithms. Alignments for which no putative paralogs were detected (labelled ‘1to1ortho’) were recorded.

#### Ambiguity-coded consensus sequences

Frequent hybridization in the eucalypts is presumed to have played a significant role in complicating efforts to resolve relationships as a fully bifurcating tree (McLay *et al.*, 2023). Given that HybPiper uses SPAdes for contig assembly, the resulting output sequences are not ambiguity coded, and only one variant is represented at each heterozygous site. Consequently, true biological relationships are less likely to be represented accurately as the frequency of heterozygous sites increases. To address this, we generated read-backed IUPAC ambiguity-coded consensus sequences for exons and introns using same the method as Kates *et al.* (2018; described at https://github.com/mossmatters/phyloscripts/tree/master/alleles_workflow). The workflow was modified only by reformatting and excluding certain scripts, and software version updates (modified scripts available at https://github.com/hkore1/TargetAllelePhasing/tree/main). This approach also incorporates intron sequences from the target loci (see https://github.com/mossmatters/HybPiper/wiki/Introns). After IUPAC-coded consensus data were assembled into exons and introns for each locus, sequences from any locus with more than one ortholog group identified by ParaGone (i.e. those that did not produce ‘1to1ortho’ files) were removed. Exon and intron sequences for each locus were then independently aligned using MAFFT v.7.505 (Katoh *et al.*, 2002; Katoh and Standley, 2013) and trimmed with trimal v.1.2rev59 (Capella-Gutierrez et al., 2009) using gap and resoverlap thresholds of 0.75, and a seqoverlap threshold of 60.

### Assembly of complete plastome sequences

To generate whole plastome sequences, we performed two rounds of assemblies on the raw reads with GetOrganelle v.1.7.7.0 (Jin *et al.*, 2020). The first round used a custom seed database (parameter -s) containing complete plastome sequences from six species (*Blakella eximia* [NC_022409.1], *Blakella maculata* [NC_022408.1], *Blakella tessellaris* [NC_022410.1], *Corymbia gummifera* [NC_022407.1], *Eucalyptus cloeziana* [NC_022388.1] and *Eucalyptus guilfoylei* [KC180798.1]). The nuclear gene target file was set as the anti-seed (-a) to exclude recruitment of nDNA reads, and we ran GetOrganelle with eight extension rounds (-R), k-mer values of 21, 65, 105 and 125 (-k), no limit on the number of reads (-max-reads INF) and the memory saving option (-memory-save).

All successfully assembled complete plastome sequences were then combined into a single multifasta file. This file was used as the new seed database for the second round of assemblies. GetOrganelle was run on previously incomplete samples using the same settings with the updated seed file. Assemblies were annotated using GeSeq v.2.03 (Tillich *et al.*, 2017).

Given that GetOrganelle was not able to assemble complete plastome sequences for multiple samples, we manually assembled partial plastome sequences for 21 species that were not represented among the set of complete GetOrganelle assemblies. Manual assembly was performed in Geneious Prime v.2023.2.1 (Biomatters Ltd), with partial plastome sequences generated by mapping reads for each sample to the most closely related (based on results of the nDNA tree) complete assembly generated by GetOrganelle as a reference (following Orel *et al*., 2023). Sites for consensus sequences were called with a 50 % threshold, and sites with fewer than four reads coverage were treated as Ns. Read mapping was manually checked, and erroneously mapped regions were replaced with 100 Ns, including 50 bp upstream and downstream of the error position. Annotations were transferred from the reference sequence and checked manually. All plastome sequences were then aligned, and sites with ≥50 % gaps were removed, along with one inverted repeat region (IRA).

### Phylogenetic analyses

Nuclear ambiguity-coded consensus alignments (nuConsensus dataset) were analysed as follows. Initially, we estimated a maximum likelihood species tree from the concatenated alignments in IQ-TREE v.2.2.2.6 (Minh *et al.*, 2020*a*), with 5000 ultrafast bootstrap (UFB; Hoang *et al.*, 2018) replicates and GENESITE resampling. The concatenated data were partitioned by locus alignment, and ModelFinder (Kalyaanamoorthy *et al.*, 2017) was used to assign models to partitions based on Bayesian information criterion scores. We then estimated gene trees for each alignment and used these to calculate gene concordance (gCF) and site concordance (sCF) factors for the concatenated species tree (Minh *et al.*, 2020*b*), sampling 1000 quartets around each branch to calculate sCF. The gene trees were used as input for analysis in ASTRAL-III v.5.7.8 (Zhang *et al.*, 2018) to estimate a species tree under the multispecies coalescent. Branches in the ASTRAL tree were annotated with normalized quartet scores (QS) and local posterior probabilities (LPP) for all three topologies. We ran ASTRAL twice, first using the raw IQ-TREE gene trees and second on the same set of gene trees after they had been put through TreeShrink v.1.3.9 (Mai and Mirarab, 2018) with default settings.

The plastome alignment was analysed in IQ-TREE v.2.2.2.6, using 5000 ultrafast bootstrap replicates and 1000 SH-aLRT (Guindon *et al.*, 2010) replicates as branch support measures. We ran two analyses; the first treated the entire plastome alignment as a single partition, and the second used ModelFinder to determine an optimal partition strategy. The second treated 50 bp blocks in the plastome alignment as partitions, and ModelFinder was used to select the best fitting partition scheme based on Bayesian information criterion scores, merging partitions using the fast relaxed clustering algorithm of PartitionFinder2, considering the top 10 % partition schemes (Lanfear *et al.*, 2014, 2017).

### Divergence time estimation

For nuclear and plastome datasets, we estimated divergence times using treePL (Smith and O’Meara, 2012). The nuclear data were subset for computational efficiency and accuracy, using SortaDate (Smith *et al.*, 2018) to identify the nuclear loci best suited for analysis. We ran gene trees generated from the nuConsensus dataset through SortaDate, filtering first by bipartition support (i.e. similarity to the species tree), second by root-to-tip variance (i.e. clock-likeness) and, finally, by tree length. The top ten loci passing these filters were selected for downstream use.

We followed the approach of Hendriks *et al.* (2023) to estimate divergence times for the nuclear data based on the topology of the ASTRAL species tree. We concatenated the ten gene alignments and ran IQ-TREE v.1.6.12 (Nguyen *et al.*, 2015) on this alignment, constraining the topology to that of the ASTRAL species tree, to infer branch lengths in substitutions per site. To assess variation in node age estimates, we generated 100 non-parametric bootstrap trees from the constrained maximum likelihood tree, allowing branch lengths to vary between replicates. We performed priming and cross-validation in treePL using the constrained maximum likelihood tree. To calibrate nodes, we used three fossils with minimum/maximum age constraints, (*Eucalyptus lynchiae* [52.71–52.00 Mya; Hermsen *et al.*, 2012], *Eucalyptus xoshemium* [52.71–52.00 Mya; Zamaloa *et al.*, 2020] and *Myrtaceidites tenuis* [50–47 Mya; Harris, 1965]) and analysed the data under two calibration schemes that differed in the node at which *M. tenuis* was applied (either at the crown of *Angophora*, *Blakella* and *Corymbia* or at the crown of *Angophora* and *Corymbia*; described in Supplementary Data Methods S1). To constrain nodes deeper than our fossil calibrations, we set a calibration at the root (i.e. the crown of *Eucalyptus* and *Angophora*/*Blakella*/*Corymbia*) with an age range of 65.0–57.2 Mya in both analyses. This range was applied conservatively, based on previously suggested divergence times; the upper limit was taken from the oldest age for that node reasoned by Ladiges *et al.* (2003), and the lower limit was taken from the youngest age estimated by Thornhill *et al.* (2019). We ran treePL on the set of 100 bootstrap trees, with the optimal parameters for each analysis identified by priming and cross-validation (Supplementary Data Methods S1). TreeAnnotator v.2.7.5 (Bouckaert *et al.*, 2019) was used to summarize mean node ages and range intervals from each set of dated bootstrap trees over the constrained maximum likelihood tree.

We estimated divergence times for the plastome dataset using the same approach, with the unpartitioned plastome tree used as the target tree. The root was calibrated in an identical manner to the nuclear analyses, and 95 % highest posterior density (HPD) interval age estimates for nodes in the dated nuclear trees were used to apply secondary calibrations at the crowns of clades shared by the nuclear and plastome trees (Supplementary Data Methods S1). We ran treePL and summarized results over the unpartitioned plastome tree with the same method used for the nuclear dating analyses.

### Characterizing the contributions of hybridization and incomplete lineage sorting to discordance

We used the plastid gene tree simulation method of García *et al.* (2017) and Folk *et al.* (2017). For computational tractability, we reduced our dataset by subsampling the nuConsensus alignments to 20 tips, including at least one sample from each major lineage, and re-estimated gene trees for each locus in IQ-TREE v.2.2.0. These gene trees were used to estimate a species tree with ASTRAL v.5.7.8. Branches of the ASTRAL species tree were scaled by a factor of four to account for organellar inheritance, and DendroPy v.4.3.0 (Sukumaran and Holder, 2010) was used to simulate 1000 gene trees under the coalescent, with the scaled ASTRAL tree as a guide tree (script available from: https://github.com/ryanafolk/tree_utilities/blob/master/simulate_gene_trees.py). After subsetting the input alignment to include only the tips present in the simulated trees, we re-estimated the empirical plastome tree in IQ-TREE for comparison with the simulated gene trees. Given that these simulated trees model only ILS (and not reticulation), relationships in the empirical plastome tree that are possible under ILS alone should also occur in the set of simulated trees. Conversely, relationships present in the empirical plastome tree and not in the simulated trees cannot be explained by ILS alone, invoking additional discordance-inducing processes. We also simulated 10 000 gene trees using the ‘simulatecoalescent’ function of the PhyloCoalSimulations package (Fogg *et al.*, 2023) in Julia v.1.9.4 (Bezanson *et al.*, 2017), which models both ILS and gene flow. Topological differences between the empirical plastome and DendroPy-simulated gene trees were visualized using ggtree::ggdensitree (Yu *et al.*, 2017) in R v.4.2.3 (R Core Team, 2023). PhyParts (Smith *et al.*, 2015) was used to assess concordance and conflict in each set of simulated trees for each node in the empirical plastome tree; outputs were summarized using the scripts ‘phypartspiecharts.py’ (from https://github.com/mossmatters/phyloscripts/blob/master/phypartspiecharts/phypartspiecharts.py) and ‘minority_report.py’ (from https://github.com/mossmatters/phyloscripts/blob/master/minorityreport/minority_report.py).

PhyloNet v.3.8.2 (Than *et al.*, 2008) was used to estimate networks from the 20-tip gene trees generated from the subsampled nuConsensus alignments. Networks were estimated using maximum pseudo-likelihood (Yu and Nakhleh, 2015), testing six maximum values for hybridization events (0–5) and performing five runs, with each run returning three networks. All inferred networks were concatenated into a single file and viewed as a consensus network (Holland and Moulton, 2003) in SplitsTree CE v.6.0.0_alpha (Huson and Bryant, 2006).

We conducted quartet sampling (Pease *et al.*, 2018; v.1.3.1) and phytop (Shang *et al.*, 2025) analyses on the nuConsensus dataset. Quartet sampling estimates quartet concordance, differential between discordant frequencies and informativeness for each branch in a species tree to provide an overview of topological confidence and consistency. Phytop uses quartet scores from ASTRAL to estimate the impact of ILS and introgression, first performing a χ^2^ test to test the goodness of fit of the MSC model at each node and subsequently calculating ILS (ILS-i) and introgression/hybridization (IH-i) indices based on quartet frequencies. For quartet sampling, we used the nuConsensus ASTRAL species phylogeny and nuConsensus alignment data as input. The alignment data were concatenated and partitioned by alignment using the ‘-genetrees’ flag, the log-likelihood threshold cut-off was set to two, and we used 100 quartet replicates per branch. For phytop, we used the nuConsensus ASTRAL species phylogeny as the input tree.

Finally, we used LDJump v.0.3.1 (Hermann *et al.*, 2019) with PhiPack (Bruen *et al.*, 2006) to estimate constant locus-wide recombination rates for each of the alignments in the nuConsensus dataset.

## RESULTS

### Phylogenetic analyses

The nuConsensus dataset included 798 alignments (399 exon and 399 intron), with a total length of 1 088 337 bp and 314 951 parsimony-informative sites. On average, alignments contained 199 sequences and were 1364 bp long, with 394 informative sites. Gene tree estimation in IQ-TREE produced 793 gene trees from these alignments (gene trees were not reconstructed for five alignments that contained at least one sequence with only gaps or ambiguities). Highly supported and concordant branches in the IQ-TREE and ASTRAL species trees were topologically congruent. Both ASTRAL species trees were topologically identical and recovered more taxonomically consistent relationships than the concatenated tree; branch support values were highest in the ASTRAL tree generated from gene trees processed with TreeShrink, hence this is discussed and presented (Fig. 2).

**Fig. 2.**
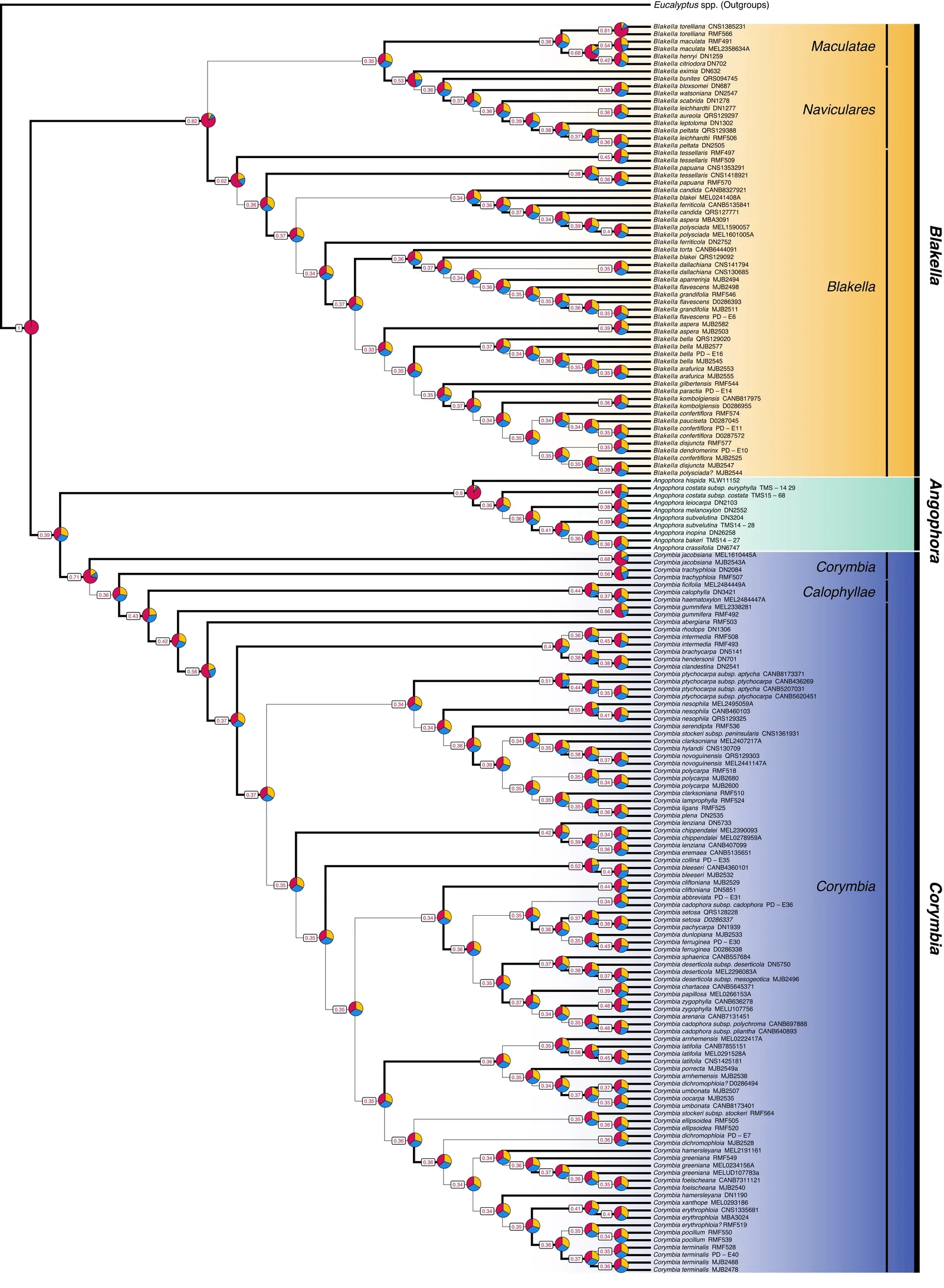
ASTRAL cladogram of *Angophora*, *Blakella* and *Corymbia*, based on 793 nuclear gene trees generated from ambiguity-coded alignments (nuConsensus) with IQ-TREE. Quartet scores for the shown topology are labelled in red at the left of nodes. Pie charts represent quartet scores for each topology at a node; red = support for the primary, shown topology; yellow/blue = support for alternative topologies. Branches with a local posterior probability of >0.8 are in bold. Section names, according to Nicolle (2022) for *Angophora* and *Corymbia* and Crisp *et al.* (2024) for *Blakella*, are shown.

*Angophora*, *Blakella* and *Corymbia* were resolved in a well-supported and highly concordant clade, within which *Angophora* and *Corymbia* were resolved as sister genera with low–moderate concordance (IQ-TREE gCF: 15 %; sCF: 39 %; ASTRAL LPP: 1; Q1S: 0.39). The monophyly of each genus was highly supported and concordant (*Angophora*, LPP: 1; Q1S: 0.9; *Blakella*, LPP: 1; Q1S: 0.82; and *Corymbia*, LPP: 1; Q1S: 0.71). Within *Blakella*, there was high support for the monophyly of sections *Maculatae* (LPP: 0.99; Q1S: 0.38), *Naviculares* (LPP: 1; Q1S: 0.53) and *Blakella* (LPP: 1; Q1S: 0.62). Species relationships within sect. *Maculatae* were well resolved and highly supported. In sections *Naviculares* and *Blakella*, relationships between species were less resolved; several well-supported clades of allied species were recovered, within which fine-scale relationships between species were unclear. Numerous species represented by multiple tips were resolved as non-monophyletic (*B. aspera*, *B. blakei*, *B. confertiflora*, *B. flavescens*, *B. grandifolia*, *B. leichardtii*, *B. papuana*, *B. peltata* and *B. tessellaris*; Supplementary Data Table S2). Within *Corymbia*, there was high support for the monophyly of sect. *Calophyllae* (LPP: 1; QS: 0.44), and sect. *Corymbia* was resolved as paraphyletic, owing to the nesting of sect. *Calophyllae* within it. Relationships in sect. *Corymbia* were somewhat equivocal, with high support (LPP: 1; QS: 0.42) for *C. gummifera* as sister to a low–moderately supported clade of the remaining species (LPP: 0.88; QS: 0.37). Within this clade, we recovered several well-supported groups of allied species, but backbone relationships and fine-scale relationships between species in these alliances were unsupported.

The plastome sequence alignment contained 131 982 sites, with 3708 parsimony-informative sites and 126 sequences. Both analyses of the plastome data produced highly resolved, topologically congruent phylogenies. Given that the partitioned (ModelFinder identified 27 optimal partitions) and unpartitioned phylogenies were essentially identical, only the tree from the unpartitioned analysis is presented and discussed (Fig. 3). Generally, branches in the plastome tree were highly supported (UFB: ≥95 %; SH-aLRT: ≥80 %). *Angophora* was resolved as monophyletic (UFB: 100 %; SH-aLRT: 100 %), and *Blakella* and *Corymbia* were resolved as polyphyletic. Two main plastid clades were resolved (Figs 3 and 4; Clades A and B); Clade A included two successive lineages from *Corymbia* (*C. gummifera* + *C. trachyphloia*, sect. *Calophyllae*), placed as sister to *Angophora* and *Blakella* sections *Naviculares*, *Maculatae* and *Blakella pro parte*. Clade B included three lineages of *B.* sect. *Blakella* nested within most of *C.* sect. *Corymbia*.

**Fig. 3.**
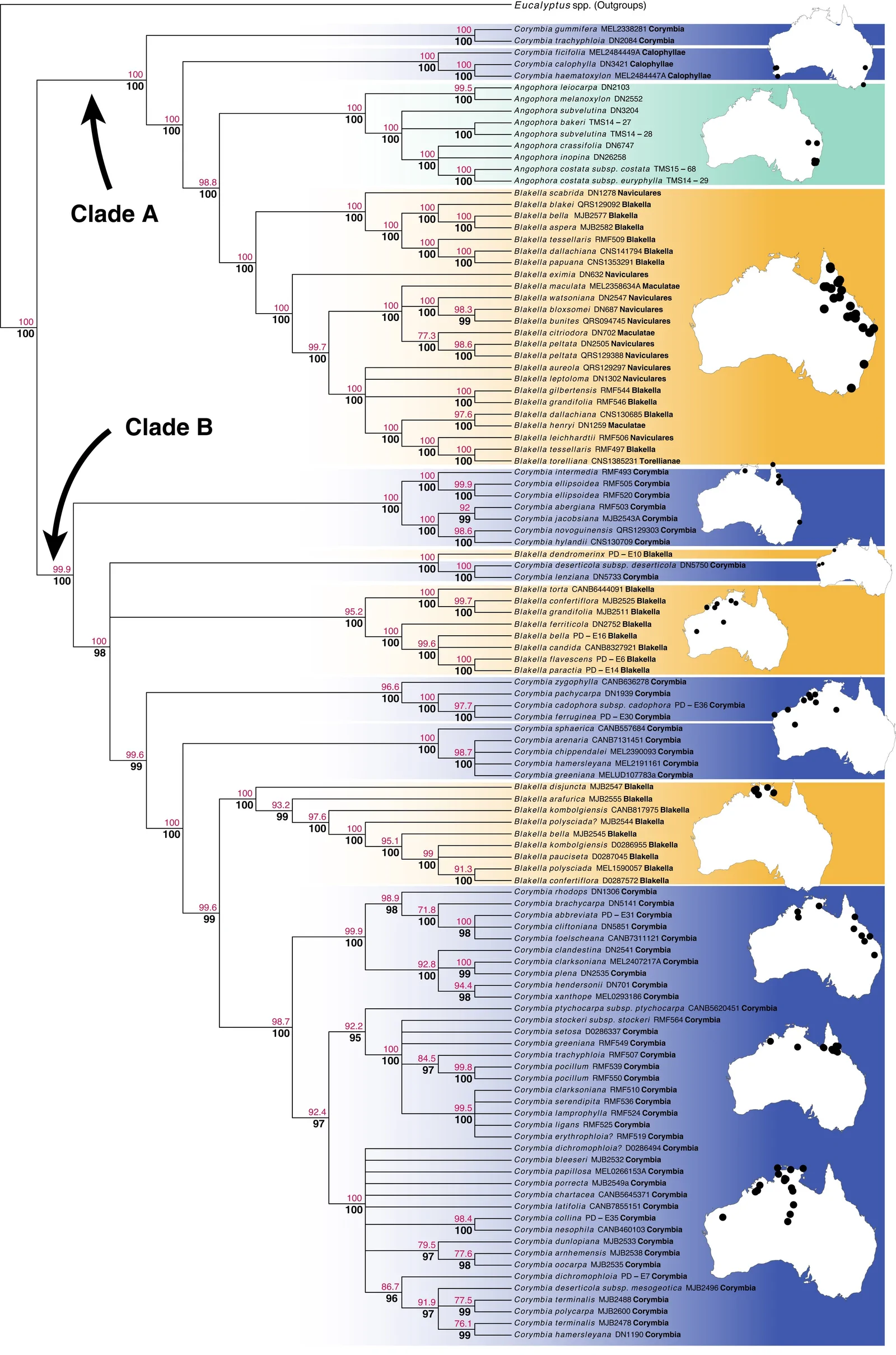
IQ-TREE maximum-likelihood cladogram of *Angophora*, *Blakella* and *Corymbia*, based on 131 982 bp plastome sequence alignment. Ultrafast bootstrap (UFboot) values are in bold below branches, and SH-aLRT values are in red above the node. Branches with <95 % UFboot support have been collapsed. Tips are labelled with section names according to Nicolle (2022) for *Angophora* and *Corymbia* and Crisp *et al.* (2024) for *Blakella*. Maps show sample collection localities for main plastid lineages.

**Fig. 4.**
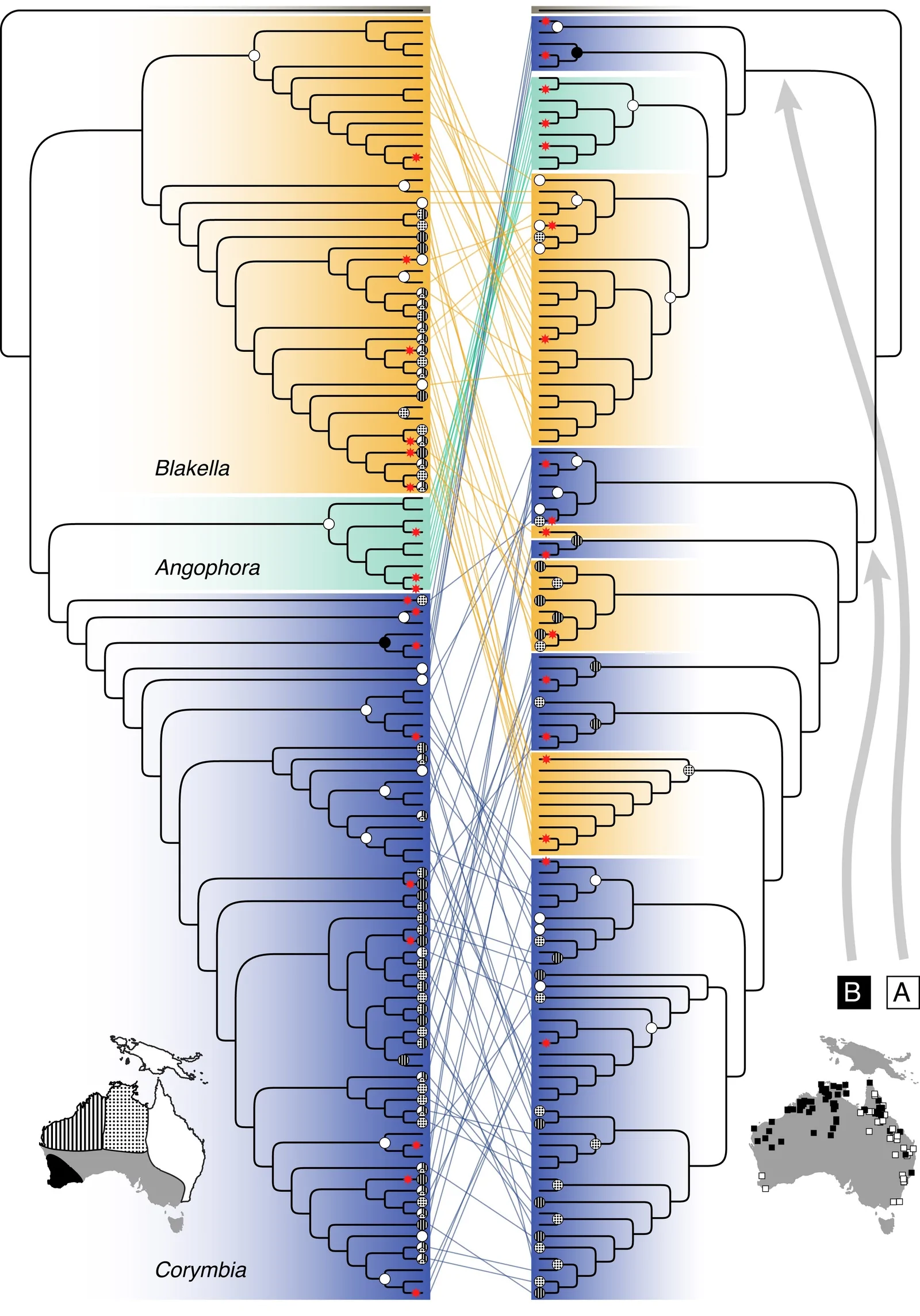
Summary tanglegram showing cytonuclear discordance between nuclear (left; Fig. 2) and plastome (right; Fig. 3) phylogenies, with tips not present in the plastome tree trimmed from the nuclear tree. The geographical occurrence of each tip is indicated by patterned pies corresponding to the areas in the map at the bottom left. For the nuclear tree, pies represent the entire geographical range of the tip. For the plastome tree, pies represent the area from which the sample was collected. Red stars indicate the 18 ingroup tips that were subsampled for incomplete lineage sorting and introgression tests; also taken were two outgroups from *Eucalyptus* (not shown). The bottom right map shows the collection localities of samples in each of the two main plastid clades, A and B (denoted by the white and black squares, respectively).

Direct comparison of the nDNA and ptDNA phylogenies revealed extensive cytonuclear discordance (Fig. 4). Relationships in the plastome tree were correlated to a greater degree with geography than with taxonomic classification; the two major clades (Clades A and B) in the plastome tree corresponded predominantly to collections made from eastern and south-western Australia (Clade A) and to samples from the Top End and the north-west of the country (Clade B) (Figs 3 and 4).

### Divergence time estimation

All alignments selected for use in the nDNA dating analyses were from introns, with a total concatenated alignment length of 17 364 bp. Node age estimates between the two calibration schemes differed minimally overall, with most nodes estimated as younger in the analysis with *M. tenuis* applied at the crown of all three genera (Fig. 5; Supplementary Data Figs S1 and S2). The largest discrepancy between analyses was the mean crown age of *Angophora* and *Corymbia* (Fig. 5; 47.0 vs. 37.7 Mya). Across the range of bootstrap trees for both analyses, age estimates for the root were consistently 65 Mya (Fig. 5). Combined minimum/maximum 95 % HPD across both analyses estimated the crown age of *Angophora*, *Blakella* and *Corymbia* between 48.7 and 47.4 Mya, the crown of *Angophora* between 13.7 and 10.2 Mya, the crown of *Blakella* between 26.6 and 23.5 Mya, and the crown of *Corymbia* between 30.6 and 24.2 Mya. Within *Blakella*, the crown of sect. *Maculatae* was estimated between 24.7 and 18.6 Mya, the crown of sect. *Naviculares* between 20.2 and 16.7 Mya, and the crown of sect. *Blakella* between 24.7 and 21.9 Mya. The crown age of *Corymbia* sect. *Calophyllae* was estimated between 21.9 and 8.5 Mya. Divergences between sister species were estimated to have occurred almost exclusively during the Miocene (∼23–5 Mya), with no sister-species divergences occurring before the Miocene and only one sister-species divergence occurring after this period (*B. dendromerinx*/*B. disjuncta*).

**Fig. 5.**
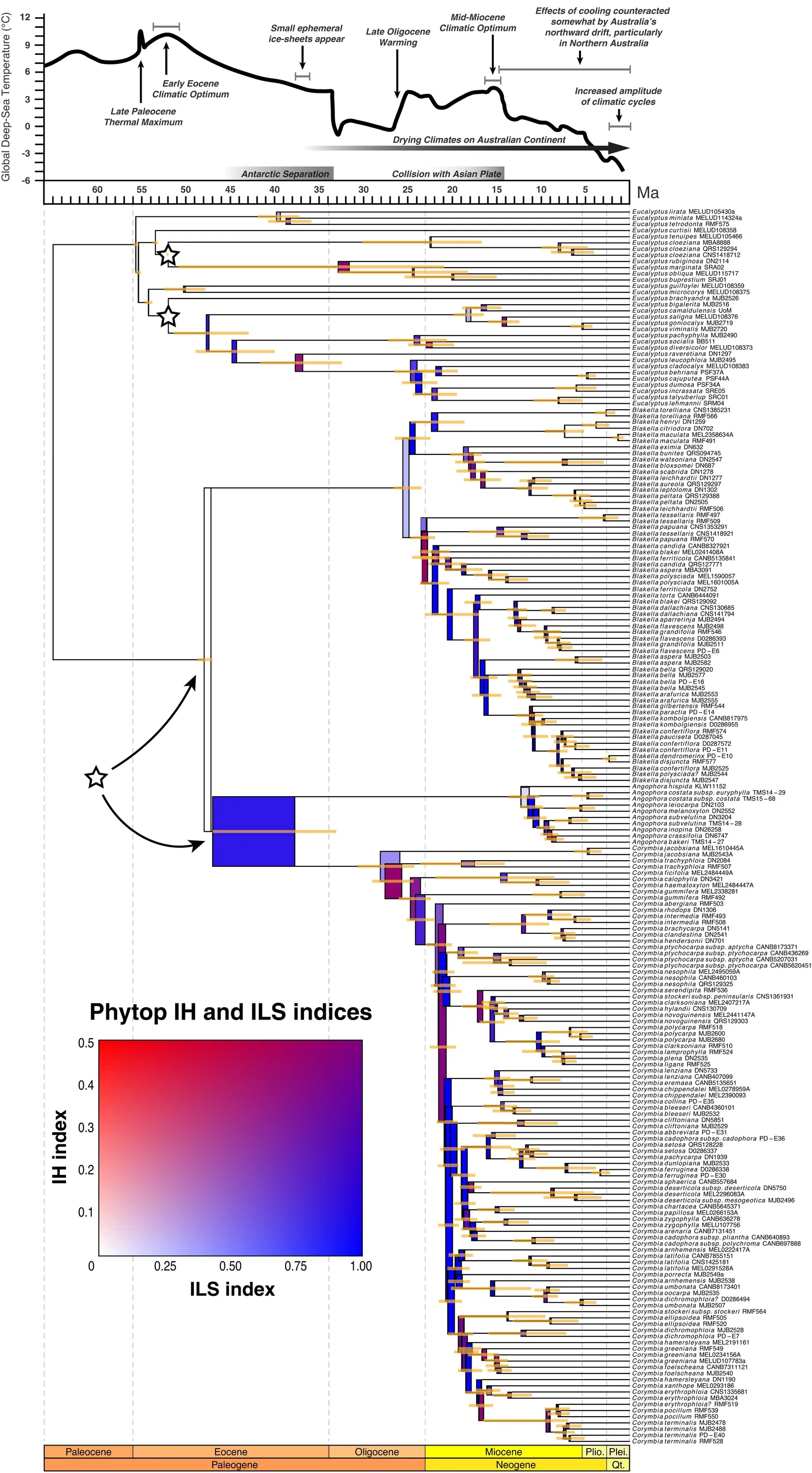
Combined time-calibrated nDNA phylogenies of *Angophora*, *Blakella* and *Corymbia*. Branches are thickened between mean node ages estimated under the two tested calibration schemes to highlight differences in results. Orange node bars are combined minimum-to-maximum 95 % HPD intervals across the two schemes. Fossil calibration points are denoted by stars at the nodes on which they were applied. Thickened portions of branches are coloured according to Phytop incomplete lineage sorting and hybridization indices (ILS = blue; IH = red); ILS strength ranges between 0 and 100 %, where 100 % represents the maximal strength (i.e. all ASTRAL quartet scores equal). IH ranges between 0 and 50 %, where 50 % represents the maximal strength (i.e. one alternative quartet score is even to that of the resolved topology). Summary of the post-Cretaceous palaeoclimate of Australia is reproduced from Byrne *et al.* (2011), originally adapted from Zachos *et al.* (2001).

Divergence time estimates for nodes in the plastome tree (Supplementary Data Fig. S3) differed greatly from those of the nDNA tree. Age estimates between the two calibration schemes mainly differed in backbone nodes of Clade B, which were ∼5 Mya older, with much larger HPD intervals, in the analysis where the crown of *Angophora*, *Blakella* and *Corymbia* was calibrated using *M. tenuis* (Supplementary Data Figs S4 and S5). Across both analyses, the root age was estimated between 65 and 61.3 Mya (95 % HPD). Crown age estimates for the two major plastid clades differed substantially, with the crown age of Clade A estimated between 46.2 and 40 Mya and the crown age of Clade B estimated between 31.4 and 10.6 Mya. The two lineages of *Corymbia* in Clade A were estimated to have diverged from the rest of the group during the Eocene, with the split of *C. trachyphloia* + *C. gummifera* occurring between 46.2 and 40.0 Mya and the split of *C.* sect. *Calophyllae* occurring between 39.0 and 28.1 Mya. In Clade A, the divergence of *Angophora* and *Blakella* was estimated between 23.8 and 18.3 Mya, with the crown age of the *Blakella* in this group estimated between 17.4 and 13.3 Mya. In Clade B, mean crown ages of the three sister lineages of *Blakella* and *Corymbia* ranged between 16.5 and 7.7 Mya across both analyses.

### Investigations of discordance and reticulation

Among the set of 1000 plastid gene trees simulated under ILS alone, relationships between *Angophora*, *Blakella* and *Corymbia* were highly discordant (Fig. 6). Almost all clades in the empirical plastome tree did not occur in the simulated trees, with the exception of shallower sister relationships between *B. peltata* and *B. blakei* (in 0.7 % of trees) and between *C. jacobsiana* and *C. ellipsoidea* (in 0.1 % of trees). Among the 798 empirical nDNA gene trees (note that gene trees were estimated for all markers because gap-only samples were not included in the subsampled alignments), proportions of shared clades with the empirical plastome species tree were only slightly higher than for the 1000 ILS-only simulated trees. Among the set of 10 000 plastid trees simulated with ILS and gene flow, two different trees contained the two major plastid clades (0.01 % of trees for each clade). Also present in this set of trees was one tree (0.01 % of trees) containing the clade *Angophora* + *B. peltata* + *B. blakei* + *C. calophylla* and 15 trees (0.15 % of trees) containing the clade *Angophora* + *B. peltata* + *B. blakei*. Clade frequencies between the three gene tree sets were most similar between the empirical nDNA gene trees and the 10 000 gene trees simulated with ILS and gene flow.

**Fig. 6.**
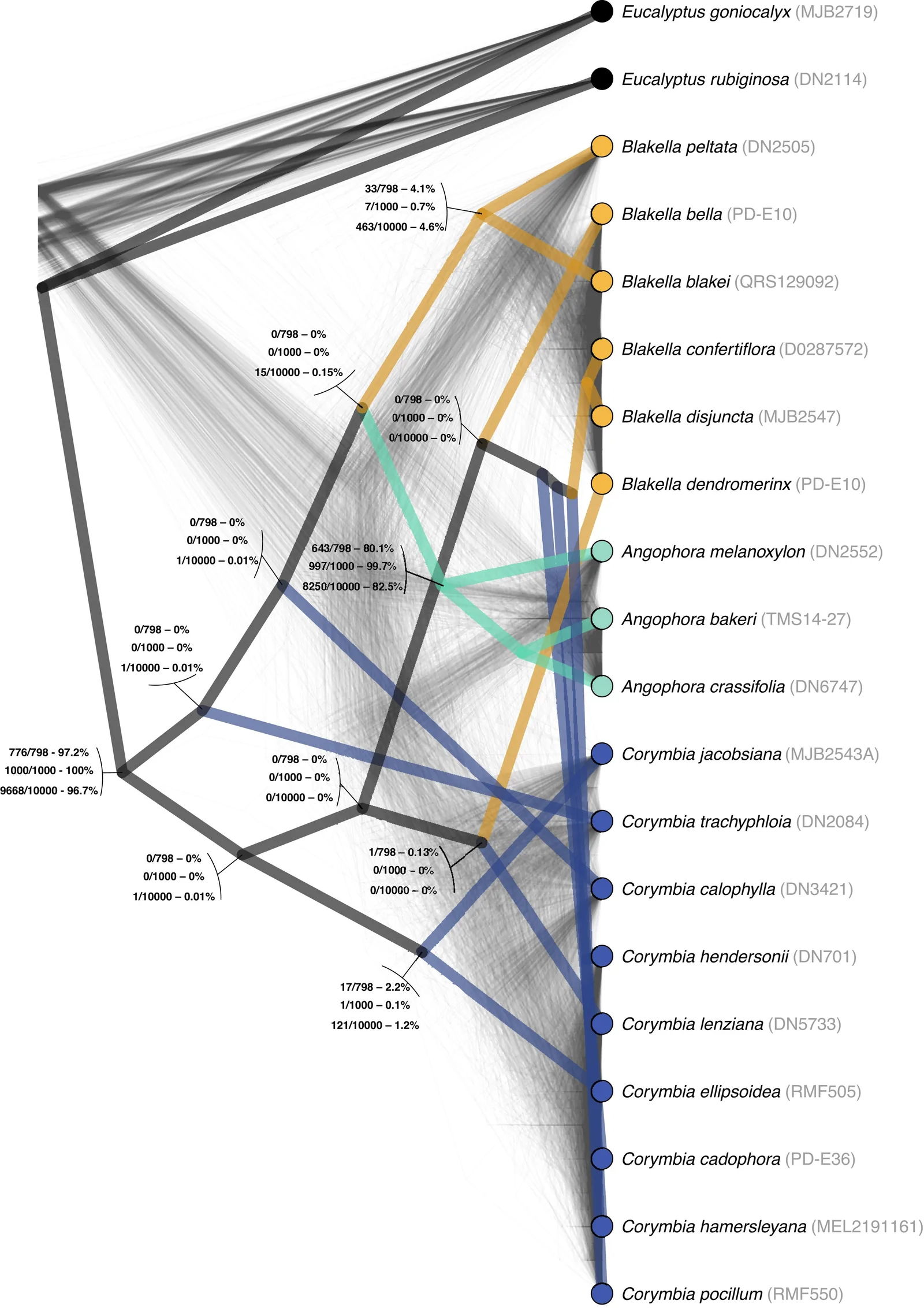
Densitree plot of 1000 subsampled ptDNA gene trees simulated under the multi-species coalescent model without gene flow using DendroPy, overlaid with the subsampled empirical plastome tree (thick branches). Displayed at deeper nodes are the clade frequencies for clades in the empirical plastome tree among 798 empirical nDNA gene trees, 1000 ptDNA gene trees simulated under the coalescent without gene flow (with DendroPy), and 10 000 ptDNA gene trees simulated under the coalescent with gene flow (with PhyloCoalSimulations).

After removing gene trees in which *Angophora*, *Blakella* and *Corymbia* were not monophyletic from the set of empirical nDNA trees and the set of 1000 ILS-only simulated trees, comparisons of the proportions of gene trees supporting each of the three possible resolutions for relationships between the genera revealed disparities between tree sets (Fig. 7). A sister relationship between *Angophora* and *Corymbia* was present in 47.5 % of simulated trees and 38.1 % of empirical nDNA trees. Among the simulated trees, the two remaining topologies were present in relatively equal frequencies (25.2 % for *Blakella* and *Corymbia* as sister; 27.1 % for *Angophora* and *Blakella* as sister), whereas the empirical nDNA trees more frequently possessed a sister relationship between *Blakella* and *Corymbia* (35.6 %) in comparison to *Angophora* and *Blakella* (26.3 %).

**Fig. 7.**
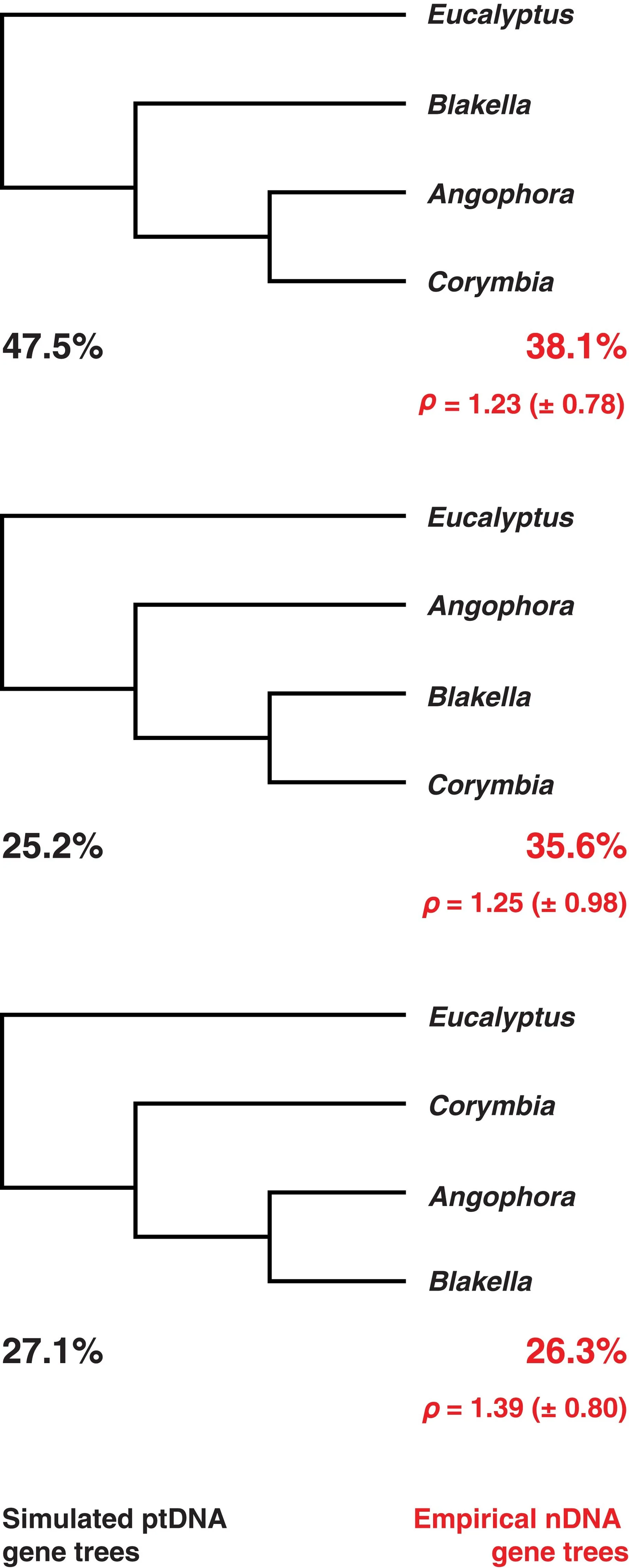
Percentage of gene trees supporting the three possible resolutions of the relationships of *Angophora*, *Blakella* and *Corymbia* under a simulated scenario with ILS only (left, black) and in empirical data (right, red). Gene trees were counted only if all three genera were monophyletic. Percentages in black are from 942 ptDNA gene trees simulated with DendroPy based on the nDNA species tree topology, which model only incomplete lineage sorting (i.e. no gene flow by introgression). Percentages in red are from 278 empirical nDNA gene trees; the value underneath is the mean (and s.d.) constant recombination rate of genes supporting the topology (*ρ*), as estimated by LDJump.

PhyloNet networks showed widespread reticulation between tips within each of the three genera and also at deeper nodes (Supplementary Data Figs S6 and S7). In particular, the networks inferred by under two or more hybridization events suggested introgression between the deepest diverging lineages in *Corymbia* (*C. calophylla*, *C. jacobsiana* and *C. trachyphloia*; Supplementary Data Fig. S7).

Quartet sampling (Supplementary Data Fig. S8) revealed generally low quartet concordance (mean: 0.07), high quartet differentials (mean: 0.78) and low–moderate quartet informativeness (mean: 0.37) across the phylogeny. Quartet fidelity scores for terminal branches were generally low (mean: 0.17). Congruent with these results, phytop revealed that, across the dataset, ILS index scores were generally high (mean ILS-i: 0.85 out of a maximum of 1), and hybridization scores were generally low (mean IH-i: 0.13 out of a maximum of 0.5) (Supplementary Data Fig. S9). LDJump failed to run for 51 of the 798 alignments in the nuConsensus dataset owing to floating point errors. Across the 747 successful alignments, the overall mean estimated recombination rate (*ρ*) was 1.45, with an average rate of 1.14 for exons and 1.80 for introns.

## DISCUSSION

Our results provide the most detailed phylogenetic assessment of *Angophora*, *Blakella* and *Corymbia* to date and are consistent with relationships recovered by Crisp *et al.* (2024). The evolutionary history of the group is deeply affected by ILS and hybridization, with ILS acting as a major driving force of conflict across the phylogeny and with hybridization impacting particular branches. These evolutionary processes have left different signatures on nuclear and plastid phylogenies; interpreting these signatures collectively and comparatively is essential for gaining insight into the shared organismal history that unites both genomic compartments. As discussed in the following sections, discordant generic relationships are best explained by ILS, with obfuscation by subsequent hybridization between *Blakella* and *Corymbia*. Topological comparisons and divergence time estimates show that nDNA and ptDNA phylogenies are temporally decoupled, revealing potential patterns of ancient plastid diversification and more recent introgression that are indicative of a complex landscape of plastid evolution. These findings provide novel insights into the evolution of a dominant group of trees in the Southern Hemisphere and the roles of discordance-inducing evolutionary processes in plants more generally.

### Summarizing phylogenomic discordance between *Angophora*, *Blakella* and *Corymbia*

#### Signatures of ILS and hybridization in nuclear genomes

Eucalypt promiscuity and the role of frequent hybridization as a source of difficulty in resolving evolutionary relationships is well discussed (Griffin *et al.*, 1988; McKinnon *et al.*, 2004; Pollock *et al.*, 2013; Larcombe *et al.*, 2015; Schuster *et al.*, 2018; Fahey *et al.*, 2022; McLay *et al.*, 2023). Despite the attention devoted to hybridization, in *Angophora*, *Blakella* and *Corymbia* records of natural intergeneric hybrids are rare (Fig. 8; Supplementary Data Table S3). There are no natural records of intergeneric hybrids between *Angophora* and any other genus (Hill and Johnson, 1995; Nicolle *et al.*, 2025), and past attempts to cross *Angophora* with species of *Blakella* have failed (Dickinson *et al.*, 2012). Natural intergeneric hybrid combinations between *Blakella* and *Corymbia* are known from only a few species: *B. maculata* × *C. gummifera* and *B. maculata* × *C. intermedia* (Hill and Johnson, 1995). Notably, these species are restricted to smaller lineages branching from the deepest splits in each genus (Fig. 8). In experimental conditions, additional intergeneric hybrid crosses have been successful between *B. torelliana* × *C. clarksoniana* and between *B. torelliana* × *C. erythrophloia* (Dickinson *et al.*, 2012). Recorded natural intersectional hybrids are more common, and even more common are hybrids within sections (Fig. 8).

**Fig. 8.**
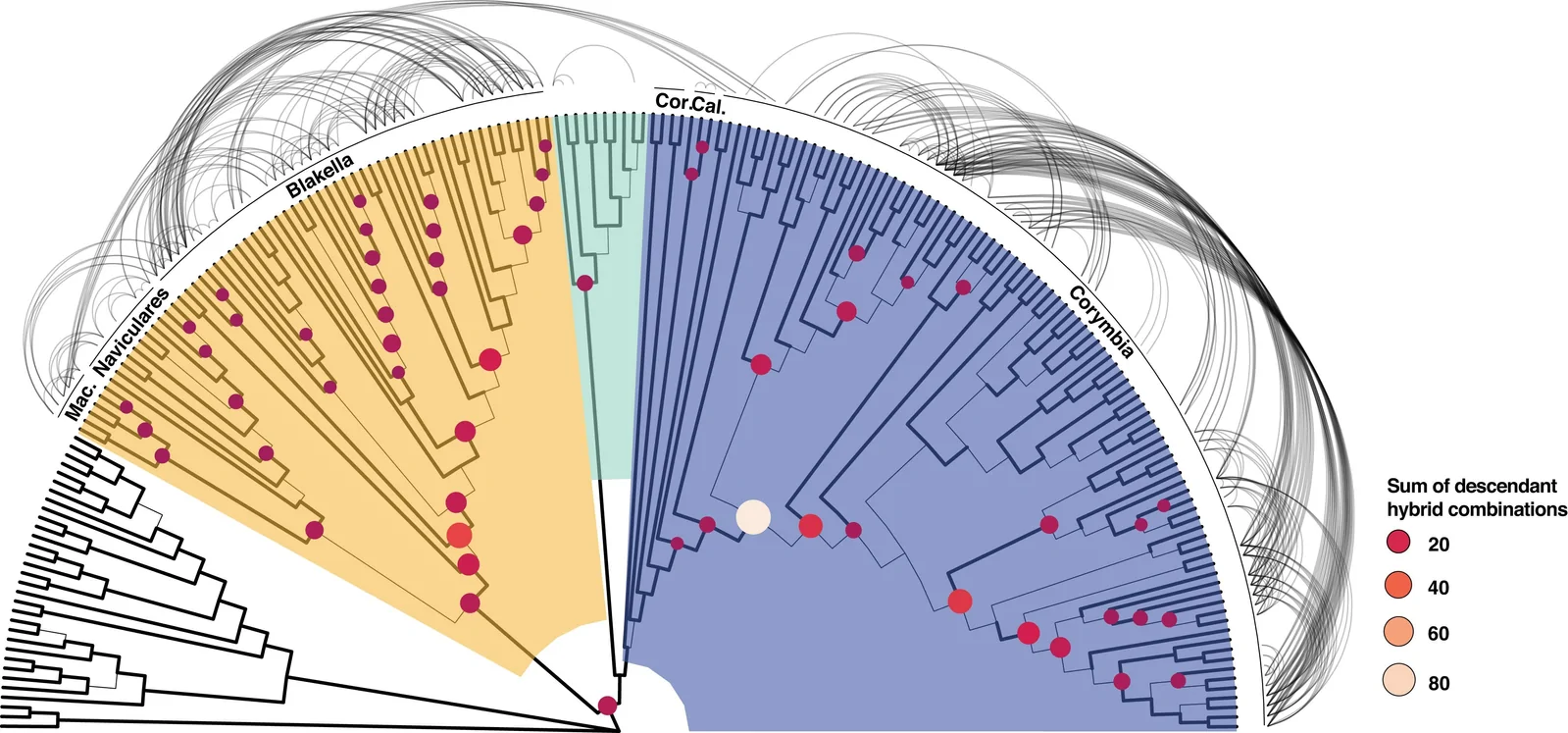
Diagram of recorded natural hybrid combinations and intergrading species in sections of *Angophora*, *Blakella* and *Corymbia*, based on data collated from Griffin *et al.* (1988) and Hill and Johnson (1995), applied to tips in the nuConsensus ASTRAL phylogeny (Fig. 2). Thick branches are those with >0.8 local posterior probability in the species tree. Points at nodes show the sum of combinations descendant from each node. Note that this count is biased by our sampling (e.g. where we have sampled four individuals of *C. terminalis*, the number of hybrid combinations between *C. terminalis* and other species will be multiplied by four). Section names, according to Nicolle (2022) for *Corymbia* and Crisp *et al.* (2024) for *Blakella*, are shown (Mac. = *Maculatae*; Cor. = *Corymbia*; Cal. = *Calophyllae*).

In contrast to hybridization, there has been less discussion of the role of ILS as a source of phylogenomic discordance in eucalypts. Recently, we showed that ILS is a major contributor to discordance in *Eucalyptus* subg. *Eudesmia* (McLay *et al.*, 2023). Our results here indicate that high levels of ILS affect almost all branches in the phylogeny of *Angophora*, *Blakella* and *Corymbia* and that this is consistent through evolutionary time (Fig. 5; Supplementary Data Fig. S10). Introgression is frequent but is concentrated at particular branches, rather than throughout the phylogeny, and always in tandem with ILS (Fig. 5; Supplementary Data Fig. S10). The complex interrelations of introgression, ILS and recombination are becoming clearer only in recent work, with genomic studies showing that ILS and introgression are positively correlated with recombination rates (Pease and Hahn, 2013; Rivas-González *et al.*, 2023; Burbrink *et al.*, 2025) and that introgression can shorten coalescence by promoting within-species variation, increasing effective population sizes and preventing the sorting of gene genealogies following speciation (Pfennig *et al*., 2016; Burbrink *et al.*, 2025). In eucalypts, linkage disequilibrium is low (Silva-Junior and Grattapaglia, 2015; Murray *et al.*, 2019) and microsynteny is highly fragmented (Ferguson *et al.*, 2023), features that reflect high recombination. Our investigations provide evidence from eucalypts that higher rates of recombination are associated with discordant ILS topologies (Fig. 7). It is likely that recombination, and both introgression and ILS, which we have shown to be pervasive through time, have contributed to phylogenomic mosaicism (Hipp *et al.*, 2020) in the group and may be associated with the very long periods required for reproductive isolation of some eucalypt species (tens of millions of years; Larcombe *et al.*, 2015) alongside life-history traits such as large population sizes (Maddison and Knowles, 2006) and long generation times (Fowler and MacMahon, 1982; Levin, 2012).

#### Patterns of discordant nuclear genomic relationships

The uncertain relationships of *Angophora*, *Blakella* and *Corymbia* based on morphological and nuclear molecular data have previously been explained by various methodological limitations or quirks (Ladiges *et al.*, 1995; Steane *et al.*, 2002; Ochieng *et al.*, 2007; Bayly *et al.*, 2008), but Crisp *et al.* (2024) were the first to demonstrate the presence of high phylogenomic discordance in nDNA data. Our results are congruent with relationships recovered by Crisp *et al.* (2024), namely *Blakella* as sister to *Angophora* and *Corymbia*, with similar levels of discordance for the sister relationship between *Angophora* and *Corymbia*. Results from quartet sampling and phytop indicate that ILS is the source of this discordance; low quartet concordance (QC: 0.04) for the branch uniting *Angophora* and *Corymbia* is moderately limited by quartet informativeness (QI: 0.44), and there is no skew in discordant alternative topologies (quartet differential, QD: 0.96) that would indicate introgression (Pease *et al.*, 2018; Fig. 5; Supplementary Data Figs S8 and S9). In other plants, rapid radiations have been invoked to explain similar QC, QI and QD scores (Pease *et al.*, 2018), and this, along with our divergence time estimates, suggests that a rapid divergence of three genera is potentially responsible for high levels of ILS (Fig. 5).

Low gene tree concordance for the sister relationship of *Angophora* and *Corymbia*, along with an unequal percentage of gene trees supporting the alternative topology with *Blakella* and *Corymbia* sister to each other (IQ-TREE gCF: 15.26 %; gDF1: 13.62 %; gDF2: 7.44 %; gDFP: 63.68 %; see also subsampled empirical trees in Fig. 7) could be taken as a signal of ancestral introgression. However, owing to the differential methods by which gene and quartet concordance metrics are calculated, we consider that this discrepancy reflects introgression between species of *Blakella* and *Corymbia* at shallower branches in the phylogeny rather than between stem branches of each genus (Minh *et al.*, 2020*b*; Lanfear and Hahn, 2024; Supplementary Data Note S1). Taken together, our results suggest that ILS is the force driving genomic conflict between the genera, with their relationships blurred further by subsequent hybridization.

At shallower phylogenetic levels, the sampling of multiple individuals for many species provides greater insight into fine-scale relationships. Localization of low statistical support, high discordance and estimates of prevalent ILS and introgression across the phylogeny has identified multiple groups, for which tree-like evolutionary histories seem unlikely and which may represent syngameons (Cervantes *et al.*, 2025). In these cases, incongruence between the current taxonomy and our nDNA phylogeny is apparent, but our sampling is insufficient to make formal taxonomic changes, because accurate delimitation requires approaches that can better reconcile reticulate evolution and genetic relationships at a very fine scale. Our results provide a phylogenomic framework for future molecular studies of such groups at the species and population levels. Groups in need of further investigation are summarized and discussed in Supplementary Data Table S2.

#### Interpreting patterns of cytonuclear discordance

Cytonuclear discordance in eucalypts is well documented in molecular phylogenetic studies, which have shown that incongruence exists between nDNA and ptDNA at shallow and deep relationships (Bayly *et al.*, 2013; Schuster *et al.*, 2018; Fahey *et al.*, 2021). Our simulations of plastid gene trees indicate that discordant relationships in the plastid phylogeny of *Angophora*, *Blakella* and *Corymbia* cannot be explained by ILS alone (Fig. 6), implying that other processes have affected plastid relationships. In other studies using the same simulation approach, this implication has been used to invoke plastid capture arising from hybridization (Folk *et al.*, 2017; García *et al.*, 2017; Lee-Yaw *et al.*, 2019; Nge *et al.*, 2021). Our extension of the method, also simulating plastid gene trees with ILS and gene flow, shows that most relationships in the plastid species tree are possible if gene flow between lineages is permitted (Fig. 6).

Despite this, in some scenarios it remains difficult to distinguish ILS and plastid capture from another potential source of cytonuclear discordance, unequal dispersal rates (i.e. migration) between pollen and seeds, which may permit the subdivision of plastome lineages whilst the nuclear genome remains panmictic (Birky *et al.*, 1983, 1989; Irwin, 2002, 2012; Kuo and Avise, 2005; Xu *et al.*, 2021). In eucalypts, pollen dispersal plays a much greater role in gene flow than seed dispersal (Potts and Wiltshire, 1997; Booth, 2017). Reported estimates of pollen-to-seed migration ratios are highly unequal compared with other plant groups (median = 17; Petit *et al.*, 2005), with pollen flow being, on average, 131.7 times greater than seed flow (median = 34.20, based on estimates for nine species by Byrne, 2008; Bloomfield *et al.*, 2011; Gauli *et al.*, 2014). This suggests that, generally, eucalypts could be more likely than other plants to experience genetic subdivision of plastomes without subdivision of nuclear genomes, a scenario that manifests in phylogenetic trees as the divergence of plastid lineages independently of divergence in the nuclear tree. Within species or hybridizing groups of species, plastid subdivision owing to unequal dispersal usually produces geographical structuring of lineages in plastid phylogenies (Petit and Excoffier, 2009; Bai *et al.*, 2010; Xu *et al.*, 2021), and traditionally, in eucalypts these patterns have been attributed to hybridization and chloroplast capture (McKinnon *et al.*, 2001; Pollock *et al.*, 2013; Fahey *et al.*, 2021). However, in the plastid phylogeny of *Angophora*, *Blakella* and *Corymbia*, the strong correspondence of Clades A and B to a geographical pattern (i.e. Clade A mainly south and Clade B mainly north of a divide running east–northeast across Australia through the Wet Tropics) probably reflects plastome diversification prior to nuclear cladogenesis (discussed in the next section).

### Temporal dynamics of discordance-inducing processes and their role in eucalypt evolution

#### Nuclear genomic insights into the timing of diversification and discordance

The primary point of comparison for our time-calibrated phylogenies is the study of Thornhill *et al.* (2019), who used a penalized likelihood approach on species trees reconstructed from a concatenated alignment of two nuclear ribosomal (ITS and ETS) and two plastid markers (*mat*K and the *psb*A–*trn*H spacer). Overlooking large topological discrepancies between our nuConsensus phylogeny and those of Thornhill *et al.* (2019) that are attributable to the use of data from different genomic compartments, node age estimates for shared clades are similar at deep nodes close to fossil calibrations, including the crown age of the clade uniting *Angophora*, *Blakella* and *Corymbia* (Table 1). Crown age estimates for those genera were generally younger than in the study by Thornhill *et al.* (2019), particularly for *Angophora* and *Blakella* (Table 1). Acknowledging that molecular dating methods rely on a suite of assumptions (Heads, 2005; Drummond *et al.*, 2006; Ho, 2014), this may be a symptom of how discordance within clades is handled, because discordant sites can be assigned incorrectly to concordant stem branches, artificially lengthening them and reducing crown age estimates (Mendes and Hahn, 2016; Carruthers *et al.*, 2022; Tiley *et al.*, 2023). However, our preferential selection of loci with gene trees most topologically similar to the species tree was intended to limit this effect (Carruthers *et al.*, 2022).

**Table 1.** Comparison of mean divergence date estimates (in millions of years ago) based on nDNA for shared major clades between this study and Thornhill *et al.* (2019).

Crown node of clade	This study (*M. tenuis* fossil placed at *Angophora*, *Blakella* and *Corymbia* crown)	This study (*M. tenuis* fossil placed at *Angophora* and *Corymbia* crown)	Thornhill *et al.* (2019)
*Eucalyptus* subgenus *Symphyomyrtus*	52 (52.2–52)	52 (52.1–52)	32.5–26.7
*Eucalyptus* subgenera *Eucalyptus*, *Idiogenes* and *Cuboidea*	52 (52–52)	52 (52–52)	50.5–40.9
*Angophora*, *Blakella* and *Corymbia*	47.2 (47.8–47.0)	48 (48.7–47.4)	47.0–46.7
*Angophora* and *Corymbia*	37.7 (41.8–33.1)	47 (47–47)	40.2–39.5
*Angophora*	11.3 (12.2–10.2)	12.3 (13.7–11.1)	46.1–26.4
*Blakella*	24.9 (26.1–23.5)	25.6 (26.6–24.0)	44.2–34.3
*Corymbia*	25.9 (27.1–24.2)	28.1 (30.6–26.0)	32.2–28.0

Ranges in parentheses indicate age 95 % HPD intervals in our study. Ages from Thornhill *et al.* (2019) are the range of point estimates for nodes across their three analyses.

Our results suggest that major diversification of extant lineages in *Blakella* and *Corymbia* occurred during the late Oligocene to early Miocene (Fig. 5). Sea level and ocean temperature variations from this period indicate that it was a time of unstable climates. At the Eocene–Oligocene boundary, there is evidence for the drastic initiation of an extended period of ocean cooling and lower sea levels (Feary *et al.*, 1991; Zachos *et al.*, 2001). Northern Australia was positioned ∼30° south of the equator, drifting north through the late Oligocene and experiencing temperate and possibly seasonal climates (Quilty, 1994; Martin, 2006; Scotese, 2016). Following this, sea levels gradually rose, and by the early Miocene ocean temperatures across northern Australia were subtropical to tropical, while central Australia had a pronounced dry season and a palynoflora reflecting vegetation adapted to seasonality (Feary *et al.*, 1991; Quilty, 1994; Martin, 2006). Deposits from this epoch indicate the presence of mosaic communities of rainforest and eucalypt forest and development of local floras in association with volcanism, offering potential opportunities for the diversification of eucalypts (Pole *et al.*, 1993; Rozefelds *et al.*, 2024). Based on macrofossil and fossil pollen data, it has been suggested that eucalypts began to expand their range throughout Australia during this period, becoming dominant across the continent in a floristic transition that was probably facilitated by increasingly dry conditions and greater fire frequency (Martin, 2006; Thornhill and Macphail, 2012; Hill *et al.*, 2016; Macphail and Thornhill, 2016). Given that community disassembly of this sort is a strong driver of rapid diversification (Levin, 2019), as are range expansions and shifts in geographical distributions (Givnish, 2010; Li and Wiens, 2022; Willi *et al.*, 2022), our results are consistent with this hypothesis. Deeper branches within *Blakella* and *Corymbia* with high signals of introgression (Fig. 5) could indicate that hybridization played some role during this early diversification and expansion, potentially by promoting adaptive innovation (Levin, 2000; Pfennig *et al.*, 2016; Buck *et al.*, 2023) as seen in extant eucalypts (Pfeilsticker *et al.*, 2022).

#### Plastid evolution is temporally decoupled from speciation but offers insight into biological processes and biogeography

Patterns of topological and temporal discordance between nuclear and plastid phylogenies imply that divergences in the plastid tree are decoupled from speciation times owing to retention of ancient plastid haplotypes and plastid capture by hybridization and introgression. Although most examples of this kind of organellar–nuclear decoupling are known from animals (Toews and Brelsford, 2012; Huang *et al.*, 2014), it has also been reported in several plant groups, including beeches (*Fagus*; Worth *et al.*, 2025), poplars (*Populus*; Huang *et al.*, 2014) and walnuts (*Juglans*; Xu *et al.*, 2021).

Comparisons of relationships and age estimates between our nuclear and plastid phylogenies demonstrate that several deep divergences in the plastid tree pre-date the crown ages of clades in the nuclear tree, implying the diversification of distinct plastid lineages before nuclear lineages and independently of them. For example, the first two divergences in plastid Clade A (i.e. of *C. gummifera* + *C. trachyphloia* and *C.* sect. *Calophyllae* from other taxa) were inferred as older (means 44.5/42.8 and 34.2/32.8 Mya, respectively) than the nDNA crown age of *Corymbia* (mean 28.1/25.9 Mya). Topologically, this pattern could be explained by plastid ILS attributable to a rapid divergence of *Angophora*, *Blakella* and *Corymbia*, with subsequent diversification of plastid lineages within *Corymbia*. However, the long internal branch lengths between the three deepest nodes in Clade A are inconsistent with rapid organismal divergence, because this would be expected to produce short internal branches among plastid lineages. In this case, branch length incongruence between nuclear and plastid trees is more likely to reflect decoupled cladogenesis between plastid and nuclear genomes, potentially attributable to unequal dispersal rates associated with skewed pollen-to-seed migration ratios. Additionally, the strong geographical structuring of lineages stemming from these deep plastid divergences better aligns with plastid subdivision from this process, because ILS would not be expected to produce predictable biogeographical patterns (Toews and Brelsford, 2012); these patterns include a clear south-eastern/north-western split corresponding to plastid Clades A and B, a south-eastern Australian group of *Corymbia* (*C. gummifera* and *C. trachyphloia*) and a south-western group of *Corymbia* (*C. calophylla*, *C. ficifolia* and *C. haemotoxylon*).

The shallow divergences of intercalated *Blakella* and *Corymbia* lineages in plastid Clade B have inferred mean ages between 16.5 and 7.7 Mya (95 % HPD; Supplementary Data Figs S3–S5), much younger than crown age estimates for the two genera based on nDNA (>20 Mya; Table 1), and are best explained by plastid capture. The age of these divergences and the fact that most of the intercalated lineages in Clade B are taxonomically constrained and include multiple representatives of either genus (instead of interspersed in a manner unaligned with their nuclear grouping) suggest that intergeneric plastid capture has been episodic rather than continuous, consistent with our nDNA estimates of hybridization through time (Fig. 5). The pattern of relationships in Clade B, which includes most of *Corymbia* (except for *C. gummifera*, *C. trachyphloia* and *C.* sect. *Calophyllae*, which are all early diverging lineages within *Corymbia* in the nDNA tree), but only three scattered clades of *B.* sect. *Blakella*, suggests that introgression has been asymmetrical, with multiple captures of *Corymbia* plastids by lineages of *B.* sect. *Blakella*. The comparatively young crown age estimates for Clade B (mean 17.2/12.0 Mya; Supplementary Data Figs S4 and S5), its distinct north-western geographical distribution and asymmetric signatures of introgression are potentially a signature of gene flow between *B.* sect. *Blakella* and *Corymbia* during past range expansions of *B.* sect. *Blakella* into the north and west, because theory suggests that genomes of invading species should be massively introgressed by those of local species if interbreeding is possible and hybrid fitness is not too low (Currat *et al.*, 2008; Excoffier *et al.*, 2009). Our nDNA dating results suggest that lineages of *Corymbia* that are presently endemic to the Top End (north-central Northern Territory; viz. *C. jacobsiana*) existed before the crown diversification of *Blakella*, with the divergences of northern and western lineages in *B.* sect. *Blakella* not occurring until after the splits of *C. jacobsiana*, *C. trachyphloia*, *B.* sect. *Maculatae* and *C. gummifera* (Fig. 5). Although our results permit a scenario where this range expansion hypothesis is possible, our sampling is insufficient to provide a robust picture of these dynamics. Nevertheless, our work provides a framework for future studies of northern Australian eucalypt biogeography that might offer further insight.

## Conclusions

Previous difficulties in resolving relationships among and within *Angophora*, *Blakella* and *Corymbia* have been attributed to various causes, and only recently has the contribution of phylogenomic discordance been identified as one of these causes. Here, we set out to generate a comprehensive phylogenetic framework for the genera and to investigate the extent of discordance and the dynamics of discordance-inducing phenomena in the evolutionary history of the group. We showed that estimates of discordance were high for almost all branches in the nuclear genome phylogeny owing to consistently high levels of ILS through time, with episodic introgression affecting particular branches. Uncertainty surrounding the relationships of *Angophora*, *Blakella* and *Corymbia* is likely to be attributable to ILS during divergence, with subsequent gene flow between *Blakella* and *Corymbia*. We showed that, at the branch concerning this relationship, nuclear genes that support ILS-affected topologies possess higher rates of recombination. The considerable cytonuclear discordance that affects the group was not attributable to ILS alone, and we suggest that, along with introgression by hybridization, sex-biased dispersal is also responsible for this type of discordance. Taken together in our time-calibrated phylogenies, patterns of both nuclear–nuclear and cytonuclear discordance reflect high levels of introgression in *Blakella* and *Corymbia*, particularly so in the latter genus, in the early stages of their diversification during the late Oligocene to early Miocene. Our work shows that discordance-inducing processes are an inherent feature of eucalypt evolution.

## SUPPLEMENTARY INFORMATION

Supplementary data are available at *Annals of Botany* online and consist of the following. Figure S1: treePL nDNA time-calibrated phylogeny with *M. tenuis* fossil applied at the crown of *Angophora* and *Corymbia*. Figure S2: treePL nDNA time-calibrated phylogeny with *M. tenuis* fossil applied at the crown of *Angophora*, *Blakella* and *Corymbia*. Figure S3: combined time-calibrated ptDNA phylogenies (Supplementary Data Figs S4 and S5) of *Angophora*, *Blakella* and *Corymbia*. Figure S4: treePL ptDNA time-calibrated phylogeny based on secondary calibrations from the nDNA chronogram with *M. tenuis* fossil applied at the crown of *Angophora* and *Corymbia*. Figure S5: treePL ptDNA time-calibrated phylogeny with *M. tenuis* fossil applied at the crown of *Angophora*, *Blakella* and *Corymbia* and secondary calibrations from the nDNA chronogram with *M. tenuis* fossil applied at the crown of *Angophora*, *Blakella* and *Corymbia*. Figure S6: SplitsTree consensus network of 18 networks inferred by PhyloNet. Figure S7: panel of all 18 networks inferred by PhyloNet. Figure S8: results of quartet sampling over the ASTRAL nuConsensus phylogeny. Figure S9 raw results from Phytop analysis of the ASTRAL nuConsensus phylogeny. Figure S10: summarized results from Phytop analysis. Methods S1: details of calibration schemes for divergence dating analyses. Note S1: interpretation of effects of differential methods of concordance factors. Table S1: list of all accessions used in the study. Table S2: details for samples representing non-monophyletic species, species of anomalous placement, or novel relationships. Table S3: recorded natural hybrid combinations in *Angophora*, *Blakella* and *Corymbia*.

## Supplementary Material

mcag242_Supplementary_Data

## Data Availability

All files used in the analyses of this study are available from Figshare (https://doi.org/10.26188/30343177). Raw sequence files are available from SRA (BioProject number: PRJNA1317555).
