# Supplementary material for: Temporal dynamics of incomplete lineage sorting, introgression and divergence in eucalypts distributed across an expansive and ancient landscape
Source: Ann Bot. 2026 Aug 5;138(5):1868–88. doi: 10.1093/aob/mcag242 (PMC13715045; doi:10.1093/aob/mcag242)
Supplement: Supplementary file 1 [file mcag242_supplementary_data.pdf]

SUPPORTING INFORMATION

Supporting Material for Orel, McLay, Fowler, Murphy, Cantrill, Bayly, "Temporal dynamics of incomplete lineage sorting, introgression and divergence in eucalypts distributed across an expansive and ancient landscape".

The following Supporting Information is available for this article:

Fig. S1 TreePL nDNA time-calibrated phylogeny with *M. tenuis* fossil applied at the crown of *Angophora* and *Corymbia*.

Fig. S2 TreePL nDNA time-calibrated phylogeny with *M. tenuis* fossil applied at the crown of *Angophora*, *Blakella* and *Corymbia*.

Fig. S3 Combined time-calibrated ptDNA phylogenies (Figs. S4 and S5) of *Angophora*, *Blakella* and *Corymbia*.

Fig. S4 TreePL ptDNA time-calibrated phylogeny based on secondary calibrations from the nDNA chronogram with *M. tenuis* fossil applied at the crown of *Angophora* and *Corymbia*.

Fig. S5 TreePL ptDNA time-calibrated phylogeny with *M. tenuis* fossil applied at the crown of *Angophora*, *Blakella* and *Corymbia* and secondary calibrations from the nDNA chronogram with *M. tenuis* fossil applied at the crown of *Angophora, Blakella* and *Corymbia*.

Fig. S6 SplitsTree consensus network of 18 networks inferred by PhyloNet.

Fig. S7 Panel of all 18 networks inferred by PhyloNet.

Fig. S8 Results of quartet sampling over the ASTRAL nuConsensus phylogeny.

Fig. S9 Raw results from Phytop analysis of the ASTRAL nuConsensus phylogeny.

Fig. S10 Summarised results from Phytop analysis.

Methods S1 Details of calibration schemes for divergence dating analyses.

Note S1 Interpretation of effects of differential methods of concordance factors.

Table S1 List of all accessions used in the study.

Table S2 Details for samples representing non-monophyletic species, species of anomalous placement, or novel relationships.

Table S3 Recorded natural hybrid combinations in *Angophora*, *Blakella* and *Corymbia*.

Fig. S1 TreePL nDNA time-calibrated phylogeny with *M. tenuis* fossil applied at the crown of *Angophora* and *Corymbia*. Node ages are the mean across 100 bootstrap trees. Node bars represent 95% HPD intervals. Mean and 95% HPD values are labelled on the right of nodes as MEAN (HPD min – HPD max). Fossil placements indicated by asterisks.

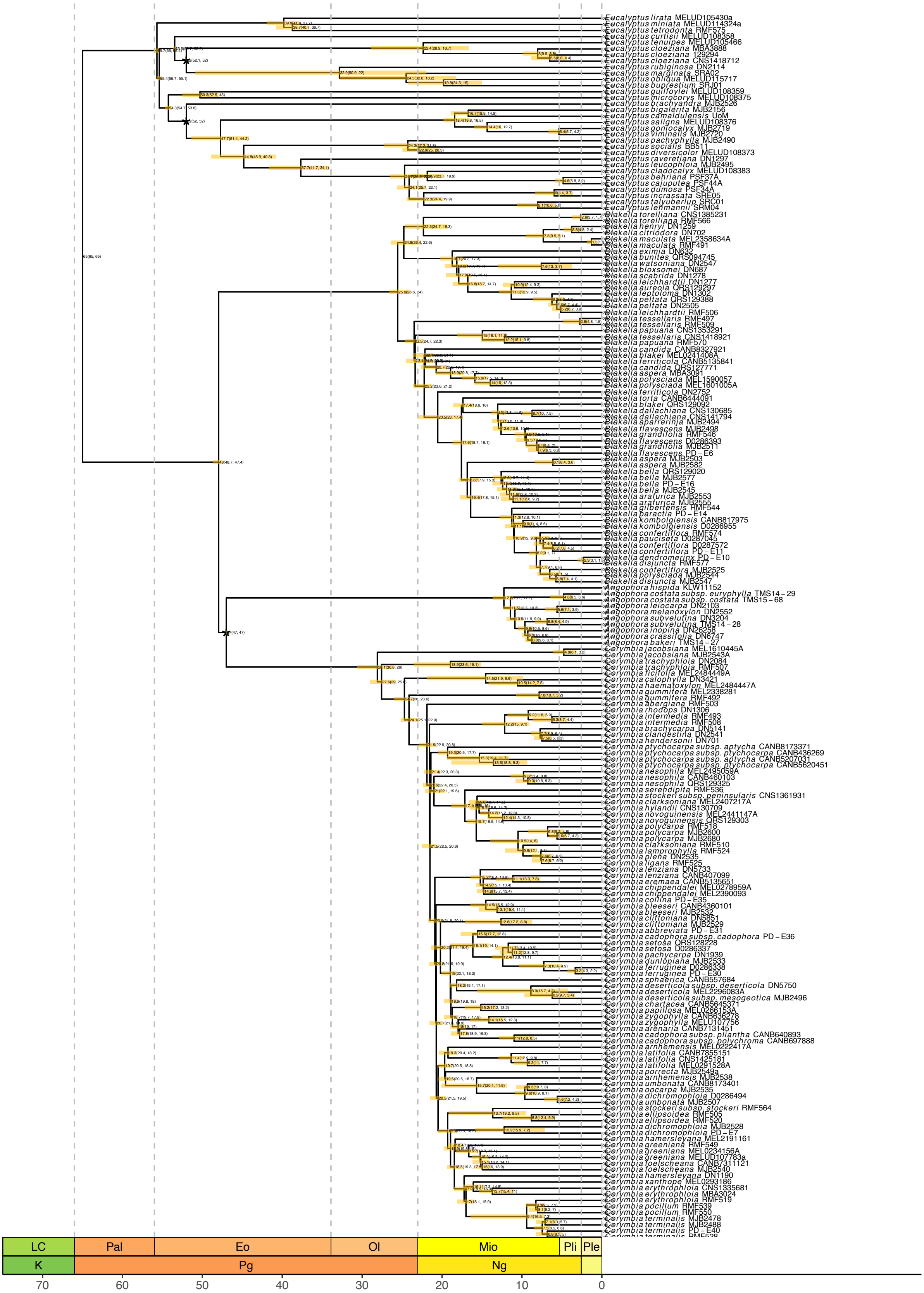

Fig. S2 TreePL nDNA time-calibrated phylogeny with *M. tenuis* fossil applied at the crown of *Angophora*, *Blakella* and *Corymbia*. Node ages are the mean across 100 bootstrap trees. Node bars represent 95% HPD intervals. Mean and 95% HPD values are labelled on the right of nodes as MEAN (HPD min – HPD max). Fossil placements indicated by asterisks.

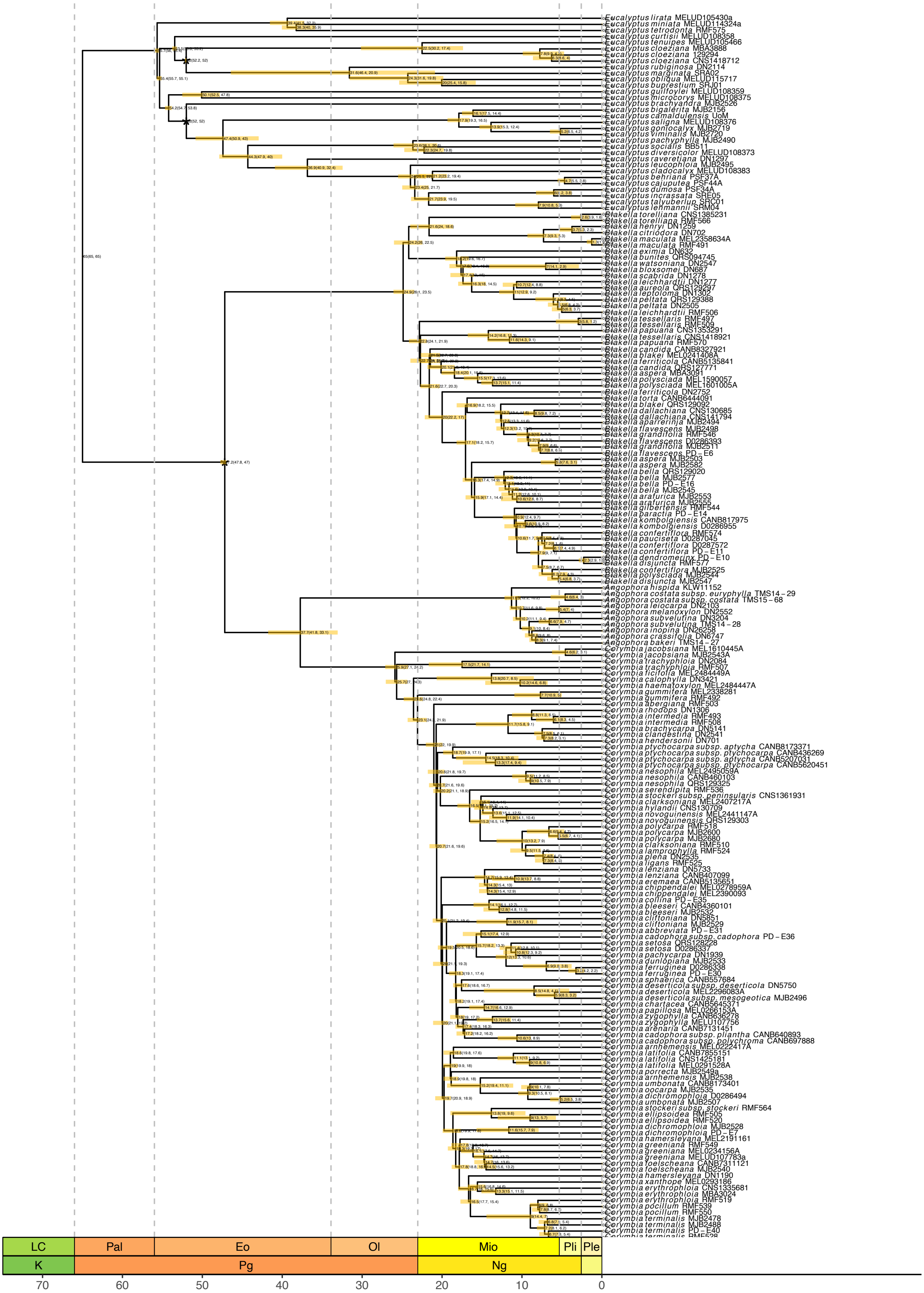

Fig. S3 Combined time-calibrated ptDNA phylogenies (i.e., Figs. S4 and S5) of *Angophora*, *Blakella* and *Corymbia*. Branches are thickened between mean node ages estimated under the two tested calibration schemes to highlight differences in results. Yellow node bars are combined 95% HPD intervals across the two schemes (i.e., minimum of both analyses to maximum of both analyses.

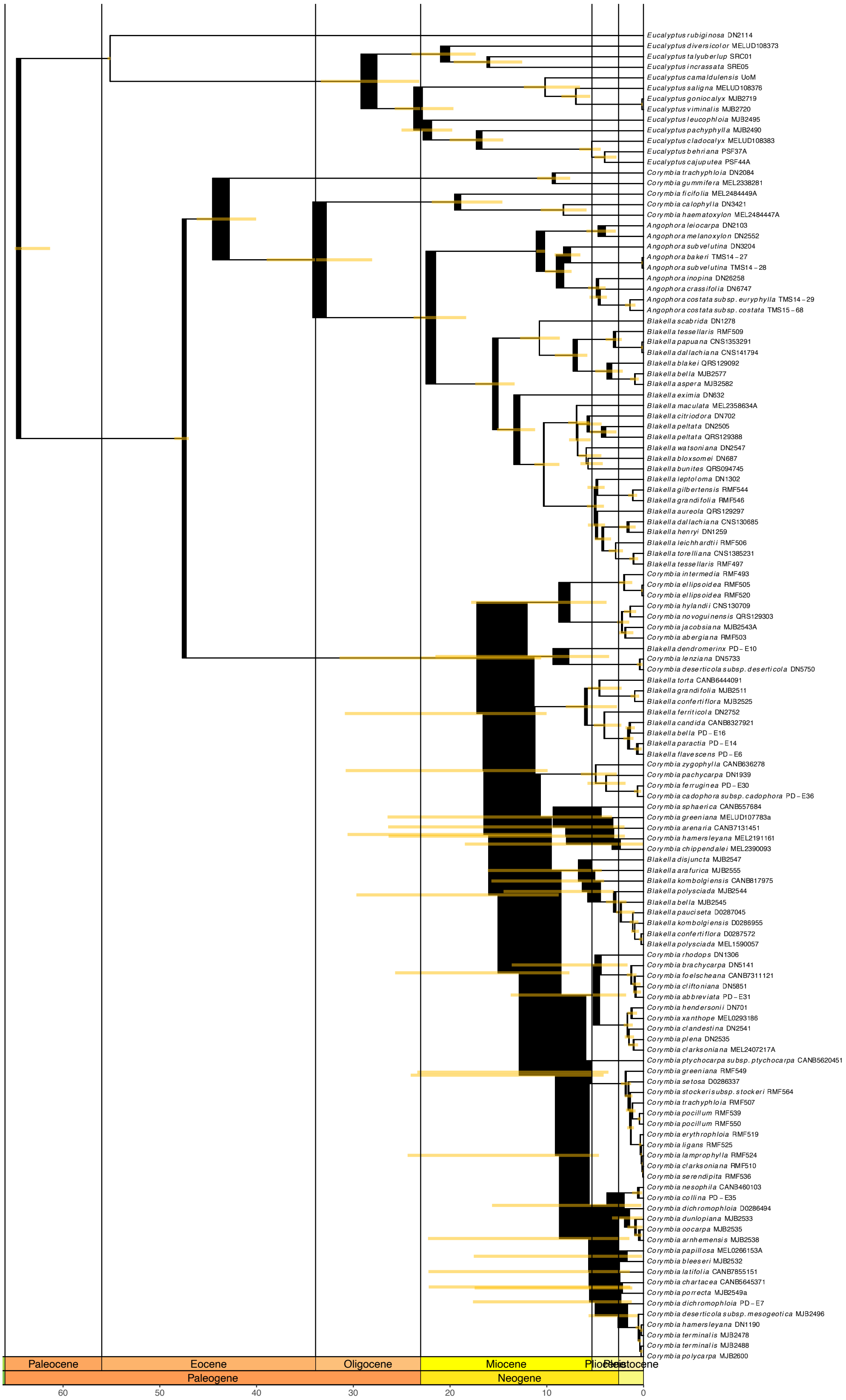

Fig. S4 TreePL ptDNA time-calibrated phylogeny based on secondary calibrations from the nDNA chronogram with *M. tenuis* fossil applied at the crown of *Angophora* and *Corymbia*. Node ages are the mean across 100 bootstrap trees. Node bars represent 95% HPD intervals. Mean and 95% HPD values are labelled on the right of nodes as MEAN (HPD min – HPD max). Calibration points indicated by asterisks.

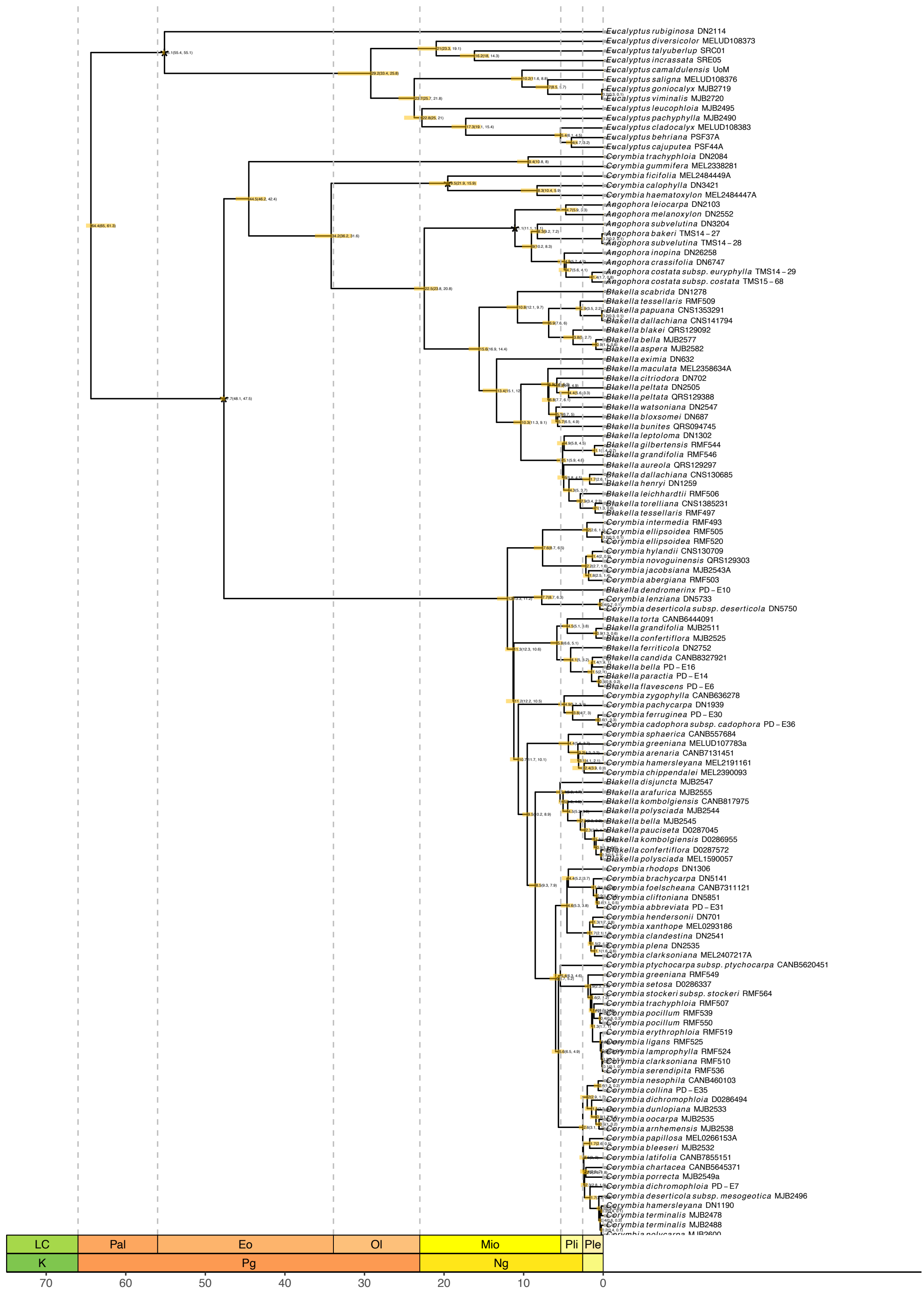

Fig. S5 TreePL ptDNA time-calibrated phylogeny with *M. tenuis* fossil applied at the crown of *Angophora*, *Blakella* and *Corymbia* and secondary calibrations from the nDNA chronogram with *M. tenuis* fossil applied at the crown of *Angophora, Blakella* and *Corymbia*. Node ages are the mean across 100 bootstrap trees. Node bars represent 95% HPD intervals. Mean and 95% HPD values are labelled on the right of nodes as MEAN (HPD min – HPD max). Calibration points indicated by asterisks.

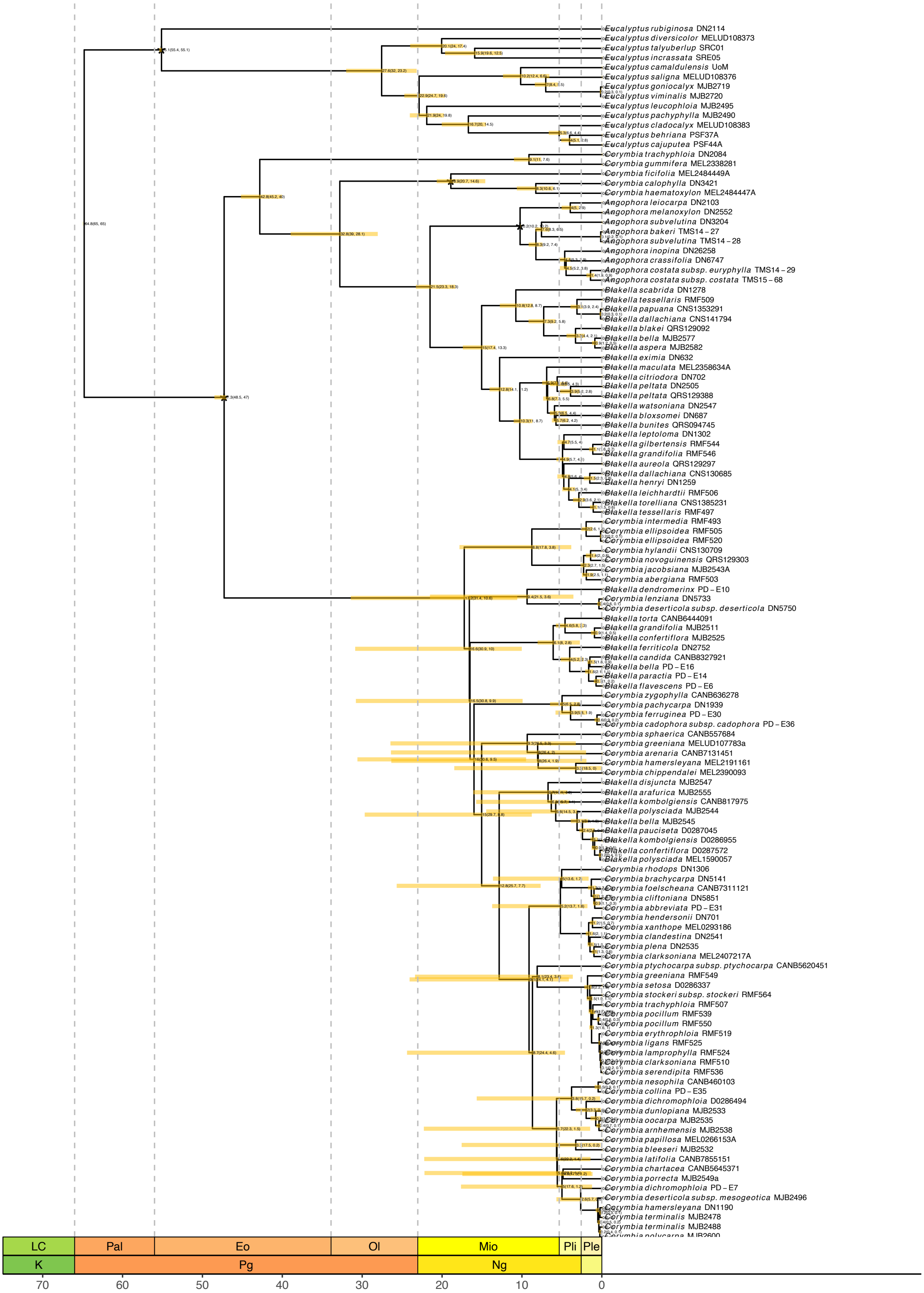

Fig. S6 SplitsTree consensus network of 18 networks inferred by PhyloNet.

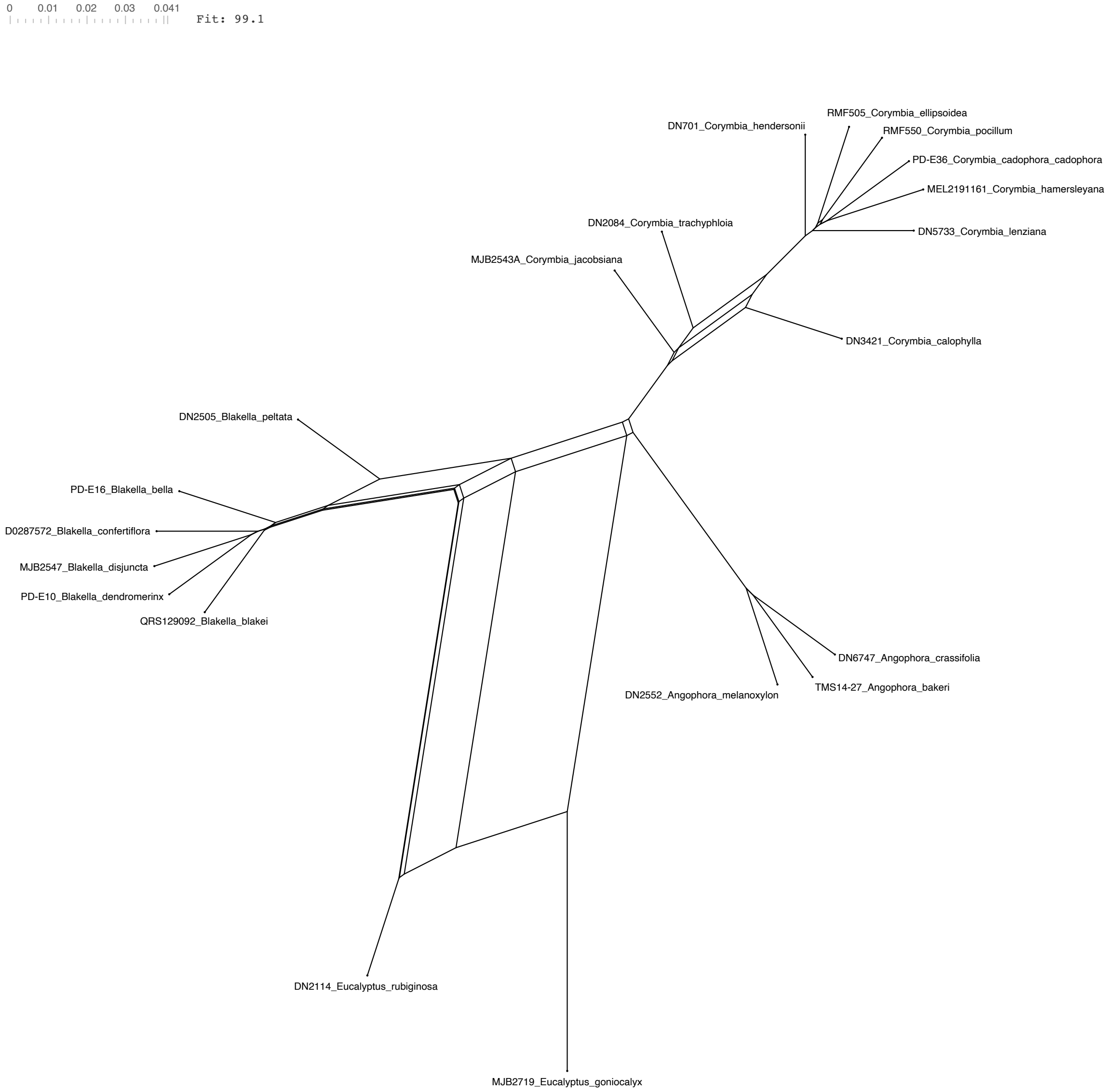

Fig. S7 Panel of all 18 networks inferred by PhyloNet. Inferred networks 1, 2 and 3 are depicted under scenarios permitting 0 to 5 hybridisation events (h0 to h5).

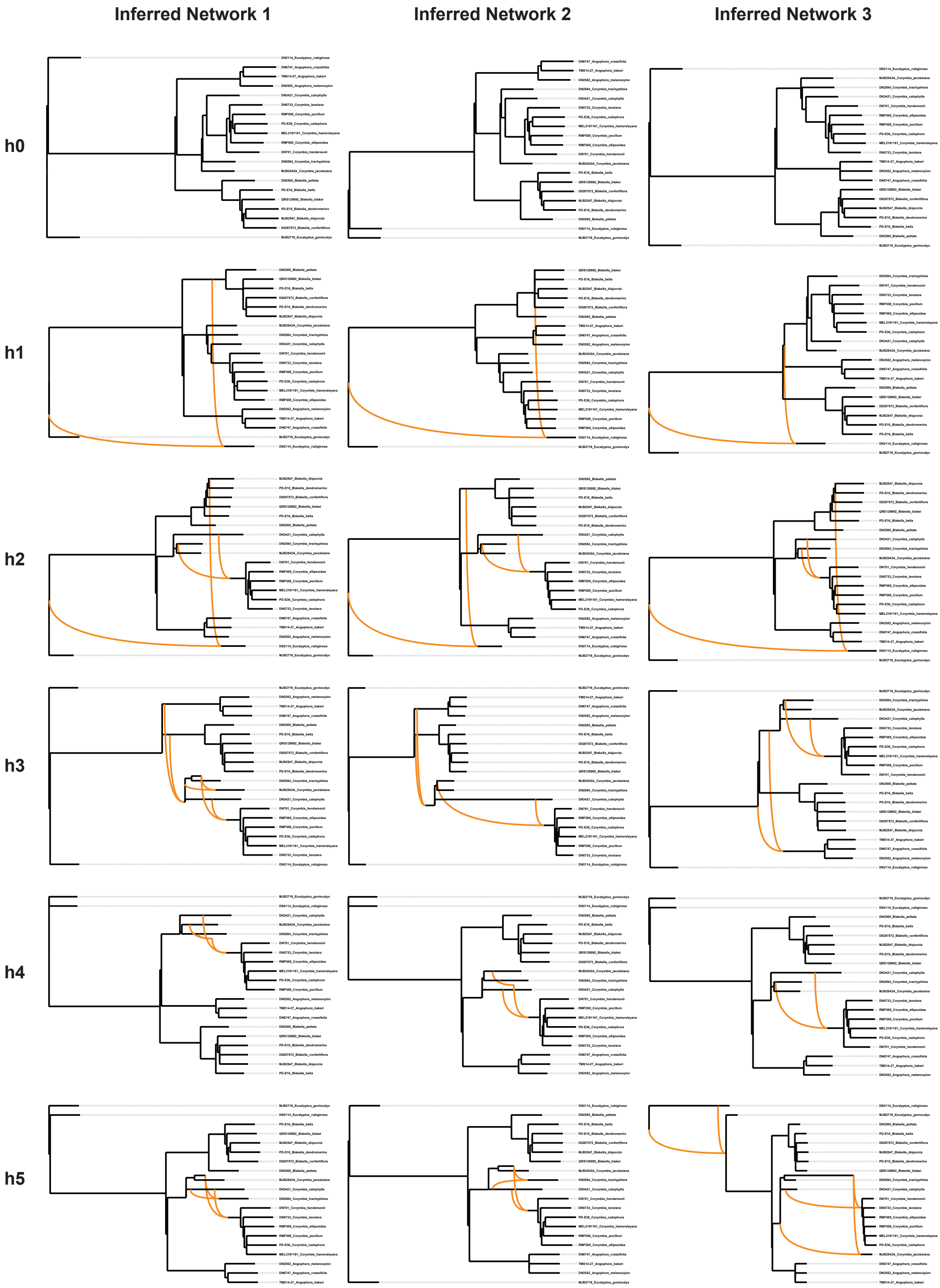

Fig. S8 Results of quartet sampling over the ASTRAL nuConsensus phylogeny. Internal branch values are Quartet Concordance/Quartet Differential/Quartet Informativeness (QC/QD/QI).

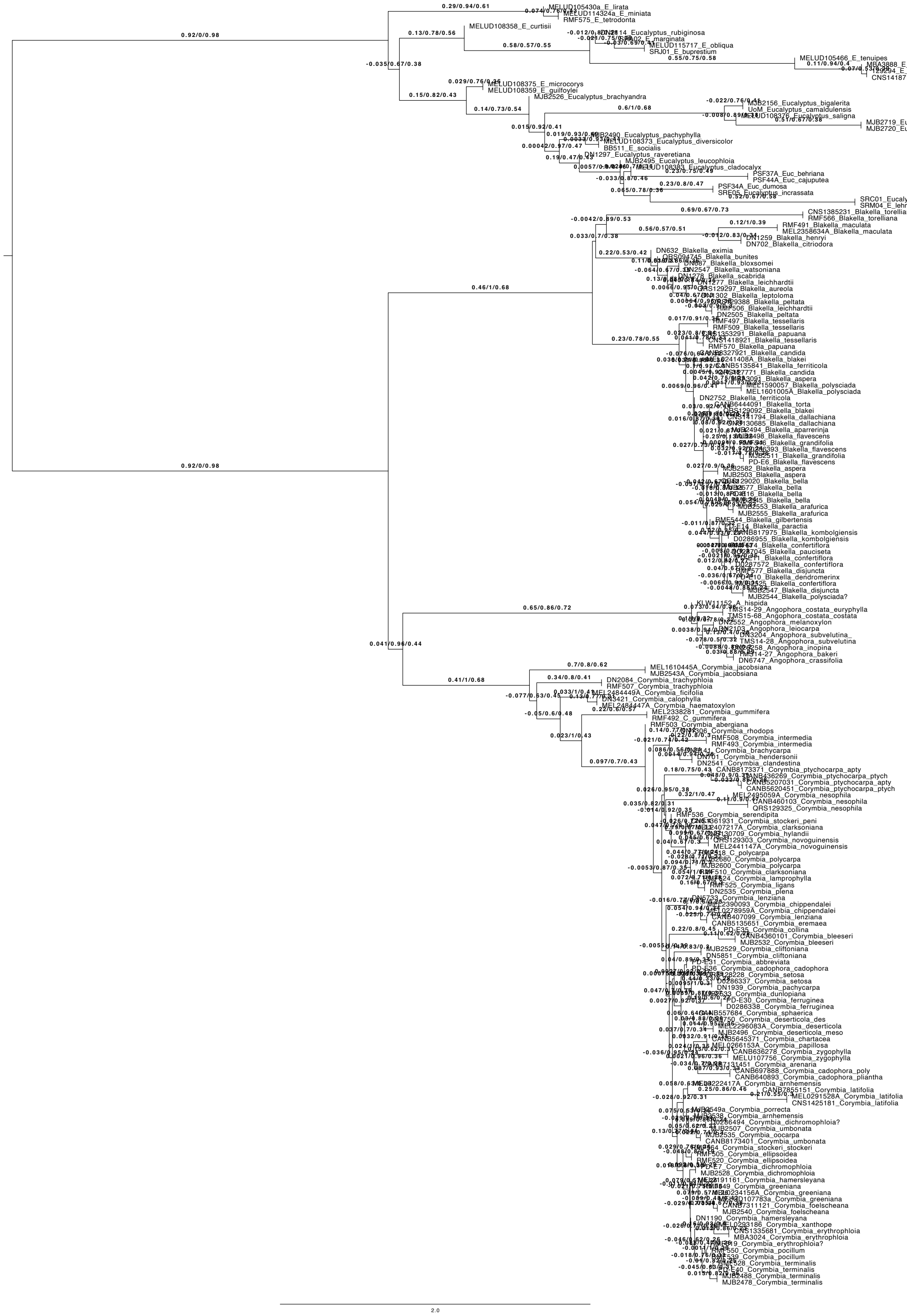

Fig. S9 Raw results from Phytop analysis of the ASTRAL nuConsensus phylogeny.

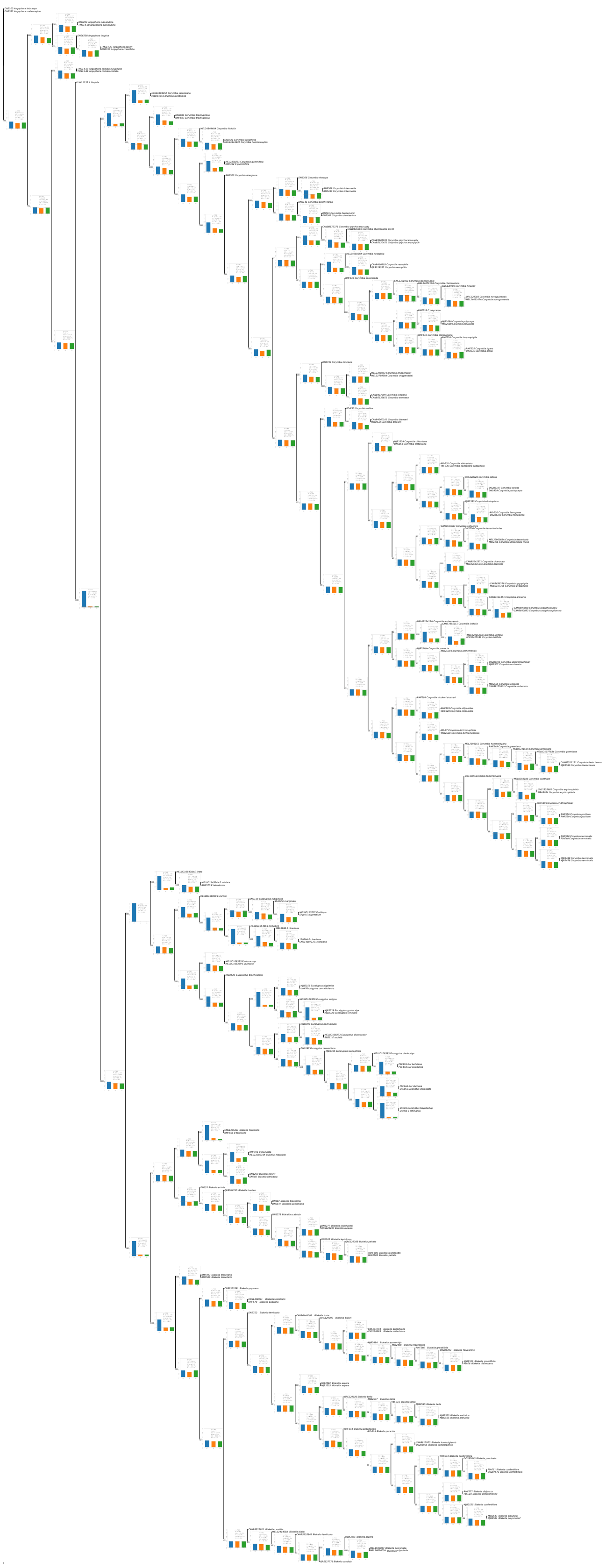

Fig. S10 Summarised results from Phytop analysis. Indices for each node each shown over the ASTRAL nuConsensus tree, with branch lengths in coalescent units. In the bottom plot, IH index values for nodes are in red and ILS index values are in blue. Fitted curves are loess smoothed conditional means.

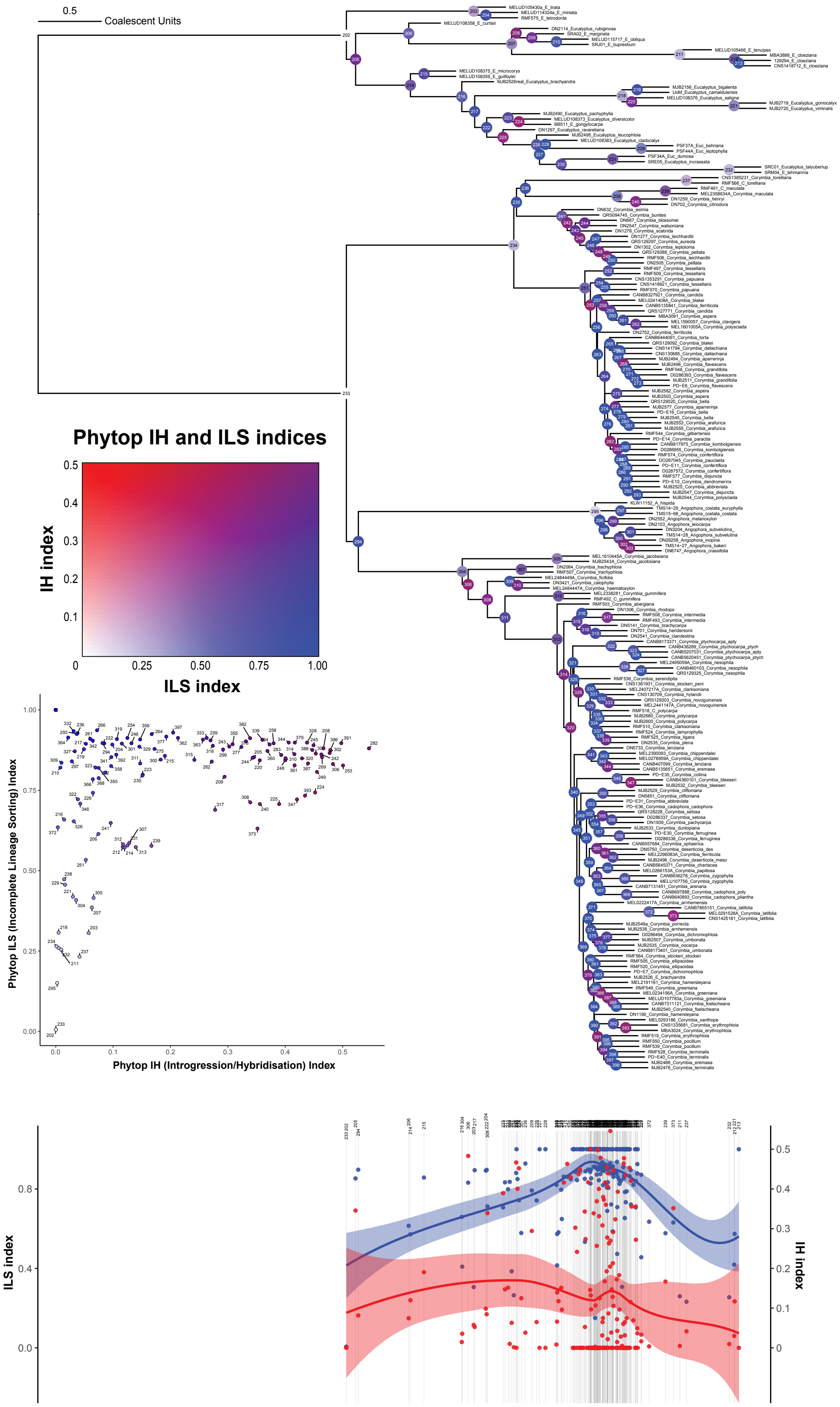

Methods S1 Details of calibration schemes for divergence dating analyses.

Two different calibration schemes were tested, which differed in the node to which the fossil pollen *Myrtaceidites tenuis* (50–47 Ma; Harris, 1965) was applied. Thornhill and Macphail (2012; doi:10.1016/j.revpalbo.2012.03.003) and Thornhill *et al.* (2012; doi:10.1016/j.ympev.2011.12.003) considered the fossil best placed at the crown of *Angophora* and *Corymbia s.l.* (i.e., including *Blakella*). However, at the time of their work, the non-monophyly of *Corymbia s.l.* was yet to be clarified. It is unclear whether their evaluation of the placement of the fossil sufficiently accounted for potential differences between pollen morphology of *Blakella* and *Corymbia*. Notably, their parsimony-optimisation approach (Thornhill *et al.*, 2012; doi:10.1016/j.ympev.2011.12.003) for testing the placement of the fossil did not include *Corymbia s.str*. Some features of extant *Angophora* and *Corymbia* pollen suggest that the fossil may be better placed at their recent common ancestor (rather than that of all three genera). These include generally larger pollen grains, with more often greatly convex sides. A preliminary reanalysis of the morphological character matrix of Thornhill *et al.* (2012), using an updated constraint tree comprised of the *Eucalyptus* clade of Crisp *et al.* (2024) joined with the *Angophora*, *Blakella* and *Corymbia* clade from this study and *Arillastrum* as an outgroup, did not provide any conclusive placement for the fossil. We therefore compared the placement of the fossil in two places: (1) at the crown of all three genera as per Thornhill *et al.* (2012), and (2) at the crown of *Angophora* and *Corymbia*.

For (1) *Eucalyptus lynchiae* (52.71–52.00 Ma; Hermsen *et al.*, 2012) was applied to the crown of *Eucalyptus* subg. *Symphyomyrtus*; *Eucalyptus xoshemium* (52.71–52.00 Ma; Zamaloa *et al.*, 2020) was applied to the crown of *Eucalyptus* subg. *Eucalyptus*, subg. *Idiogenes* and subg. *Cuboidea*; and *Myrtaceidites tenuis* (50–47 Ma; Harris, 1965) was applied to the crown of *Angophora*, *Blakella*, *Corymbia*. Following priming and cross-validation, treePL was run with 'opt' and 'optad' set to 2, 'optcvad' set to 4, and with the 'moredetail' and 'moredetailad' options enabled. Based on cross-validation, the smoothing parameter was set to 1×10^{-7}.

For (2) *Eucalyptus lynchiae* (52.71–52.00 Ma; Hermsen *et al.*, 2012) was applied to the crown of *Eucalyptus* subg. *Symphyomyrtus*; *Eucalyptus xoshemium* (52.71–52.00 Ma; Zamaloa *et al.*, 2020) was applied to the crown of *Eucalyptus* subg. *Eucalyptus*, subg. *Idiogenes* and subg. *Cuboidea*; and *Myrtaceidites tenuis* (50–47 Ma; Harris, 1965) was applied to the crown of *Angophora* and *Corymbia*. Following priming and cross-validation, treePL was run with 'opt' and 'optad' set to 2, 'optcvad' set to 4, and with the 'moredetail' and 'moredetailad' options enabled. Based on cross-validation, the smoothing parameter was set to 1×10^{-12}.

For the plastid dating analyses, there were four clades shared by both nuclear and plastid tree topologies that were useful for calibrating nodes in the plastid trees. We conducted two analyses based on results from the two nuclear analyses:

(1) was based on ages in the nuclear tree with *M. tenuis* placed at the crown of *Angophora*, *Blakella* and *Corymbia* (scheme 1), we set one fossil calibration (*M. tenuis*) at the crown of *Angophora, Blakella and Corymbia* (50–47 Ma) and three secondary calibrations at: the

crown of *Eucalyptus* subg. *Eucalyptus*, subg. *Alveolata*, subg. *Cruciformes* and subg. *Symphyomrytus* (55.71–55.10 Ma), the crown of *Angophora* (12.19–10.19 Ma) and the crown of *C.* sect. *Calophyllae* (20.67–8.51 Ma). Following priming and cross-validation, treePL was run on the bootstrap trees with the values for 'opt' and 'optad' set to 2, 'optcvad' set to 4, the 'moredetailad' option enabled, and a smoothing parameter of 1×10^{-10}.

(2) was based on ages in the nuclear tree with *M. tenuis* placed at the crown of *Angophora* and *Corymbia* (scheme 2), we set four secondary calibrations at: the crown of *Angophora, Blakella and Corymbia* (48.70–47.49 Ma), the crown of *Eucalyptus* subg. *Eucalyptus*, subg. *Alveolata*, subg. *Cruciformes* and subg. *Symphyomrytus* (55.72–55.09 Ma), and the crowns of *Angophora* (13.67–11.09 Ma) and *C.* sect. *Calophyllae* (21.89–9.88 Ma). Following priming and cross-validation, treePL was run on the bootstrap trees with the values for 'opt' and 'optad' set to 1, 'optcvad' set to 4, the 'moredetail' and 'moredetailad' options enabled, and a smoothing parameter of 1×10^{-8}.

Note S1 Interpretation of effects of differential methods of concordance factors.

Because the approach for calculating gene concordance employed in IQ-TREE samples every tip descendant from the branch of interest, it is more sensitive to effects caused by outlying discordant samples than quartet-based approaches, which only sample four tips around the branch of interest. For example, this is known to make IQ-TREE gene concordance disproportionally affected by gene tree estimation error compared to site and quartet concordance metrics, as trivial non-monophyly (i.e., when a single tip jumps between clades around the branch of interest) reduces gene concordance. Similarly, any tips containing a signal of introgression between descendant clades at shallower nodes will always be sampled, and consequently this signal will always be picked up. In contrast, this signal of introgression is only picked up by quartet concordance approaches if a sampled tip for a quartet around the branch of interest happens to be introgressed. Thus, quartet concordance measures are dictated by the proportion of tips around the branch of interest that possess a signal of introgression, rather than the collective signal of introgression displayed by all tips in the clades descendant from the branch which is conveyed by IQ-TREE gene concordance.

Table S1 List of all accessions used in the study. Asterisks denote samples that were removed from the study before analysis, but for which data were generated.

Taxon	Study ID Number	Herbarium/Living Collection Voucher Number	Collector Number	Collection Latitude	Collection Longitude	NCBI SRA Accession Number
Angophora bakeri	TMS14-27	MEL 2484394A	TMS14-27	-33.68	150.77	SRR35291023
Angophora costata subsp. costata	TMS15-68	CANB 552649.1	TMS15-68	-33.80	150.60	SRR35291019
Angophora costata subsp. euryphylla	TMS14-29	MEL 2484396A	TMS14-29	-33.04	150.68	SRR35291021
Angophora crassifolia	DN6747	Currency Creek Arboretum Dried Collection	DN6747	-33.77	151.24	SRR35291131
Angophora hispida	KLW11152	NSW1054512	KLW11152	-34.14	151.11	SRS17943861
Angophora inopina	DN26258	Currency Creek Arboretum Living Collection	DN6258	-33.19	151.49	SRR35291137
Angophora leiocarpa	DN2103	AD 163292	DN2103	-28.15	151.56	SRR35291145
Angophora melanoxylon	DN2552	AD 163739	DN2552	-27.96	148.69	SRR35291138
Angophora subvelutina	DN3204	Currency Creek Arboretum Living Collection	DN3204	-33.84	150.65	SRR35291135
Angophora subvelutina	TMS14-28	MEL 2484395A	TMS14-28	-33.63	150.77	SRR35291022
Blakella aparrerinja	MJB2494	MELUD114292a	MJB2494	-20.28	134.23	SRR35291086
Blakella arafurica	MJB2553	MELUD114349a	MJB2553	-12.66	131.32	SRR35291109
Blakella arafurica	MJB2555	MELUD114351a	MJB2555	-12.87	131.72	SRR35291108
Blakella aspera	MBA3091	MBA 3091.1	Fox, I.D. 1075	-17.80	138.14	SRR35291055
Blakella aspera	MJB2503	MELUD114302a	MJB2503	-18.19	133.73	SRR35291127
Blakella aspera	MJB2582	MELUD142140a	MJB2582	-20.69	139.54	SRR35291084
Blakella aureola	QRS129297	QRS 129297.1	Connors, J.R. 1307	-22.21	148.01	SRR35291192
Blakella bella	MJB2545	MELUD114341a	MJB2545	-13.54	131.39	SRR35291112
Blakella bella	MJB2577	MELUD142139a	MJB2577	-18.73	138.49	SRR35291107
Blakella bella	PD-E16	MELUD127759a	PD-E16	-17.75	122.27	SRR35291077
Blakella bella	QRS129020	QRS 129020.2	Gray, B. 9088	-20.54	140.41	SRR35291194
Blakella blakei	MEL0241408A	MEL 0241408A	Forster, P.I. 18740	-26.45	143.78	SRR35291051
Blakella blakei	QRS129092	QRS 129092.1	Connors, J.R. 1261	-22.78	142.30	SRR35291193
Blakella bloxsomei	DN687	AD 162445	DN687	-26.49	150.76	SRR35291060
Blakella bunites	QRS094745	QRS 94745.1	Brooker, M.I.H. 10453	-23.80	149.17	SRR35291072
Blakella candida	CANB8327921	CANB 832792.1	Latz, P. 30589	-23.12	128.70	SRR35291173
Blakella candida	QRS127771	QRS 127771.2	Hyland, B. 16825	-23.38	119.77	SRR35291196
Blakella citriodora	DN702	AD 162591	DN702	-24.22	148.23	SRR35291058
Blakella confertiflora	D0287572	DNA D0287572	Buckley, K. s.n.	-14.54	132.27	SRR35291157
Blakella confertiflora	MJB2525	MELUD114322a	MJB2525	-15.38	131.61	SRR35291123
Blakella confertiflora	PD-E11	MELUD127754a	PD-E11	-15.79	128.73	SRR35291079
Blakella confertiflora	RMF574	MELUD127977a	RMF574	-13.91	143.18	SRR35291027
Blakella dallachiana	CNS130685	CNS 130685.1	Connors, J.R. 1263a	-20.62	144.40	SRR35291172

Blakella dallachiana	CNS141794	CNS 141794.1	Mcdonald, K.R. 15000	-14.08	143.62	SRR35291165
Blakella dendromerinx	PD-E10	MELUD127753a	PD-E10	-17.68	123.09	SRR35291116
Blakella disjuncta	MJB2547	MELUD114343a	MJB2547	-13.18	131.10	SRR35291111
Blakella disjuncta	RMF577	MELUD127980a	RMF577	-13.79	143.13	SRR35291026
Blakella eximia	DN632	AD 162956	DN632	-33.39	150.98	SRS17943860
Blakella ferriticola	CANB5135841	CANB 513584.1	Connors, J.R. 989	-26.16	125.16	SRR35291141
Blakella ferriticola	DN2752	AD 163641	DN2752	-26.36	118.41	SRR35291136
Blakella flavescens	D0286393	DNA D0286393	Luxton, S. 3327	-17.98	133.62	SRR35291160
Blakella flavescens	MJB2498	MELUD114296a	MJB2498	-19.27	134.18	SRR35291128
Blakella flavescens	PD-E6	MELUD127749a	PD-E6	-17.91	125.32	SRR35291069
Blakella gilbertensis	RMF544	MELUD127947a	RMF544	-18.22	142.78	SRR35291034
Blakella grandifolia	MJB2511	MELUD114310a	MJB2511	-16.31	133.43	SRR35291125
Blakella grandifolia	RMF546	MELUD127949a	RMF546	-18.24	142.29	SRR35291033
Blakella henryi	DN1259	AD 99624267	DN1259	-27.44	152.20	SRR35291155
Blakella kombolgiensis	CANB817975	CANB 817975.1	Westaway, J. 4438	-12.46	134.90	SRR35291175
Blakella kombolgiensis	D0286955	DNA D0286955	Lewis, D.L. 3531	-14.01	136.65	SRR35291158
Blakella leichhardtii	DN1277	AD 99624246	DN1277	-24.15	147.88	SRR35291153
Blakella leichhardtii	RMF506	MELUD127909a	RMF506	-17.38	145.26	SRR35291183
Blakella leptoloma	DN1302	AD 99624227	DN1302	-19.01	146.10	SRR35291149
*Blakella leptoloma**	QRS095012	QRS 95012.1	Brooker, M.I.H. 10407	-19.02	146.10	SRR35291197
Blakella maculata	MEL2358634A	MEL 2358634A	Jeanes, J.A.; Rule, K. 2795	-37.63	148.22	SRR35291103
Blakella maculata	RMF491	MELUD127894a	RMF491	-35.37	150.40	SRS17943922
Blakella papuana	CNS1353291	CNS 135329.1	Mcdonald, K.R. 8657	-14.46	143.68	SRR35291169
Blakella papuana	RMF570	MELUD127973a	RMF570	-15.43	144.42	SRR35291028
Blakella paractia	PD-E14	MELUD127757a	PD-E14	-17.90	122.30	SRR35291078
Blakella pauciseta	D0287045	DNA D0287045	Brennan, K. 3562	-13.91	136.70	SRR35291057
Blakella peltata	DN2505	AD 163709	DN2505	-18.62	143.83	SRR35291143
Blakella peltata	QRS129388	QRS 129388.1	Connors, J.R. 1281	-20.38	145.63	SRR35291188
*Blakella petalophylla**	DN1262	AD 99624268	DN1262	-25.43	150.96	SRR35291154
Blakella polysciada	MEL1590057	MEL 1590057A	Evans, M. 3543	-14.47	132.33	SRR35291046
Blakella polysciada	MEL1601005A	MEL 1601005A	Evans, M. 3472	-14.63	132.68	SRR35291045
Blakella polysciada?	MJB2544	MELUD114340a	MJB2544	-13.54	131.39	SRR35291113
Blakella scabrida	DN1278	AD 99624296	DN1278	-24.36	147.39	SRR35291151
Blakella tessellaris	CNS1418921	CNS 141892.1	Addicott, E.P. 3040	-14.49	143.83	SRR35291164
Blakella tessellaris	RMF497	MELUD127900a	RMF497	-26.64	153.09	SRR35291186
Blakella tessellaris	RMF509	MELUD127912a	RMF509	-17.44	145.38	SRR35291180
Blakella torelliana	CNS1385231	CNS 138523.1	Dowe, J.L. 1149	-16.87	145.68	SRR35291167
Blakella torelliana	RMF566	MELUD127969a	RMF566	-16.87	145.59	SRS17943929
Blakella torta	CANB6444091	CANB 644409.1	Slee, A.V. 4565	-16.49	126.21	SRR35291106

Blakella watsoniana	DN2547	AD 163668	DN2547	-24.63	149.11	SRR35291139
*Blakella watsoniana**	QRS094748	QRS 94748.1	Brooker, M.I.H.10447	-25.62	148.33	SRR35291198
Corymbia abbreviata	PD-E31	MELUD127774a	PD-E31	-15.92	128.14	SRR35291074
Corymbia abergiana	RMF503	MELUD127906a	RMF503	-17.39	145.37	SRR35291185
Corymbia arenaria	CANB7131451	CANB 713145.1	Barrett, R.L. 3779	-15.98	125.32	SRR35291063
Corymbia arnhemensis	MEL0222417A	MEL 0222417A	Menkhorst, K.A. 988	-12.63	133.43	SRR35291054
Corymbia arnhemensis	MJB2538	MELUD114334a	MJB2538	-14.18	132.19	SRR35291117
Corymbia bleeseri	CANB4360101	CANB 436010.1	Hill, K. 4095	-14.45	127.37	SRR35291199
Corymbia bleeseri	MJB2532	MELUD114328a	MJB2532	-14.65	132.11	SRR35291124
Corymbia brachycarpa	DN5141	Currency Creek Arboretum Dried Collection	DN5141	-20.77	145.04	SRR35291133
Corymbia cadophora subsp. cadophora	PD-E36	MELUD127779a	PD-E36	-16.90	125.77	SRR35291071
Corymbia cadophora subsp. pliantha	CANB640893	CANB 640893.1	Slee, A.V. 4536	-15.96	126.92	SRR35291118
Corymbia cadophora subsp. polychroma	CANB697888	CANB 697888.1	Barrett, R.L. 3610	-16.60	128.30	SRR35291075
Corymbia calophylla	DN3421	PERTH 5788722	DN3421	-32.27	116.51	SRR35291134
Corymbia chartacea	CANB5645371	CANB 564537.1	Nicolle, D. 4214	-12.17	133.54	SRR35291093
Corymbia chippendalei	MEL0278959A	MEL 0278959A	Latz, P.K. 13585	-24.58	132.50	SRR35291048
Corymbia chippendalei	MEL2390093	MEL 2390093A	Gibson, N. 6497	-25.08	120.74	SRR35291102
Corymbia clandestina	DN2541	AD 163750	DN2541	-22.68	147.39	SRR35291140
Corymbia clarksoniana	MEL2407217A	MEL 2407217A	PIF39480	-27.94	151.22	SRR35291101
Corymbia clarksoniana	RMF510	MELUD127913a	RMF510	-17.46	145.37	SRR35291179
Corymbia cliftoniana	DN5851	AD 241364	DN5851	-17.50	128.38	SRR35291061
Corymbia cliftoniana	MJB2529	MELUD114326a	MJB2529	-15.61	131.12	SRR35291121
Corymbia collina	PD-E35	MELUD127778a	PD-E35	-16.87	125.82	SRR35291073
Corymbia deserticola	MEL2296083A	MEL 2296083A	Walsh, N.G. 6491	-22.72	117.76	SRR35291042
Corymbia deserticola subsp. deserticola	DN5750	AD 241351	DN5750	-24.32	116.84	SRR35291130
Corymbia deserticola subsp. mesogeotica	MJB2496	MELUD114294a	MJB2496	-20.02	134.21	SRR35291041
Corymbia dichromophloia	MJB2528	MELUD114325a	MJB2528	-15.61	131.12	SRS17943913
Corymbia dichromophloia	PD-E7	MELUD127750a	PD-E7	-17.17	125.35	SRR35291068
Corymbia dichromophloia?	D0286494	DNA D0286494	Luxton, S. 3397	-16.99	134.03	SRR35291159
Corymbia dunlopiana	MJB2533	MELUD114329a	MJB2533	-14.40	132.36	SRR35291120
Corymbia ellipsoidea	RMF505	MELUD127908a	RMF505	-17.37	145.31	SRR35291184
Corymbia ellipsoidea	RMF520	MELUD127923a	RMF520	-18.20	144.57	SRR35291040
Corymbia eremaea	CANB5135651	CANB 513565.1	Connors, J.R. 969	-26.00	128.13	SRR35291152
Corymbia erythrophloia	CNS1335681	CNS 133568.1	Pettit, N. s.n.	-21.02	146.13	SRR35291170
Corymbia erythrophloia	MBA3024	MBA 3024.1	Addicott, E. 1108	-19.32	143.69	SRR35291056
Corymbia erythrophloia?	RMF519	MELUD127922a	RMF519	-18.18	144.63	SRR35291176
Corymbia ferruginea	D0286338	DNA D0286338	Jobson, P.C. 3295	-17.67	133.62	SRR35291161
Corymbia ferruginea	PD-E30	MELUD127773a	PD-E30	-15.90	127.32	SRR35291076
Corymbia ficifolia	MEL2484449A	MEL 2484449A	TMS15-31	-35.03	116.88	SRR35291098

Corymbia foelscheana	CANB7311121	CANB 731112.1	Brennan, K. 9918	-14.22	134.57	SRR35291189
Corymbia foelscheana	MJB2540	MELUD114336a	MJB2540	-14.18	132.19	SRR35291115
Corymbia greeniana	MEL0234156A	MEL 0234156A	Brooker, M.I.H. 11486	-13.08	132.67	SRR35291053
Corymbia greeniana	MELUD107783a	MELUD107783a	Ladiges, P.Y. s.n.	-17.73	123.65	SRR35291094
Corymbia greeniana	RMF549	MELUD127952a	RMF549	-17.90	141.26	SRR35291032
Corymbia gummifera	MEL2338281	MEL 2338281A	Jeanes, J.A.; Walsh, N.G. 2665	-37.61	149.72	SRR35291104
Corymbia gummifera	RMF492	MELUD127895a	RMF492	-35.32	150.46	SRS17943924
Corymbia haematoxylon	MEL2484447A	MEL 2484447A	TMS15-29	-32.40	116.00	SRR35291099
Corymbia hamersleyana	DN1190	AD 99625487	DN1190	-22.72	117.76	SRR35291156
Corymbia hamersleyana	MEL2191161	MEL 2191161A	Slee, A.V.; Connors, J.R. 4479	-22.12	114.07	SRR35291043
Corymbia hendersonii	DN701	AD 162442	DN701	-24.22	148.24	SRR35291059
Corymbia hylandii	CNS130709	CNS 130709.1	Connors, J.R. 1439	-15.66	144.58	SRR35291171
Corymbia intermedia	RMF493	MELUD127896a	RMF493	-31.77	152.67	SRR35291187
Corymbia intermedia	RMF508	MELUD127911a	RMF508	-17.43	145.40	SRR35291181
Corymbia jacobsiana	MEL1610445A	MEL 1610445A	Hill, K.; Stanberg, L. 3934	-12.75	135.56	SRR35291044
Corymbia jacobsiana	MJB2543A	MELUD114339a	MJB2543A	-13.75	131.74	SRR35291114
Corymbia lamprophylla	RMF524	MELUD127927a	RMF524	-18.79	144.60	SRR35291039
Corymbia latifolia	CANB7855151	CANB 785515.1	Crisp, M.D. 10550	-15.70	126.38	SRR35291178
Corymbia latifolia	CNS1425181	CNS 142518.1	Mcdonald, M.W. 1314	-8.83	141.25	SRR35291163
Corymbia latifolia	MEL0291528A	MEL 0291528A	Cowie, I.D. 8350	-12.65	135.21	SRR35291047
Corymbia lenziana	CANB407099	CANB 407099.1	Brooker, M.I.H. 10720	-26.08	120.13	SRR35291200
Corymbia lenziana	DN5733	AD 241349	DN5733	-24.95	114.94	SRR35291129
Corymbia ligans	RMF525	MELUD127928a	RMF525	-19.09	145.19	SRR35291038
Corymbia nesophila	CANB460103	CANB 460103.1	Leach, G.J.; Dunlop, C.R. 3941	-11.79	130.51	SRR35291166
Corymbia nesophila	MEL2495059A	MEL 2495059A	Leach, G.J. 4810	-14.01	126.19	SRR35291097
Corymbia nesophila	QRS129325	QRS 129325.1	Connors, J.R. 1431	-12.66	142.06	SRR35291190
Corymbia novoguinensis	MEL2441147A	MEL 2441147A	Wannan, B.S. 3785	-10.14	142.31	SRR35291100
Corymbia novoguinensis	QRS129303	QRS 129303.1	Connors, J.R. 1432	-11.39	142.41	SRR35291191
*Corymbia novoguinensis**	QRS094673	QRS 94673.1	Fell, D.G. & Delaney, M.2232	-13.63	142.67	SRR35291064
Corymbia oocarpa	MJB2535	MELUD114331a	MJB2535	-14.32	132.43	SRR35291119
Corymbia pachycarpa	DN1939	PERTH 5218012	DN1939	-18.35	127.56	SRR35291147
Corymbia papillosa	MEL0266153A	MEL 0266153A	Cowie, I.D. 6534	-11.90	136.45	SRR35291049
Corymbia plena	DN2535	AD 163670	DN2535	-20.77	145.03	SRR35291142
Corymbia pocillum	RMF539	MELUD127942a	RMF539	-18.29	143.53	SRR35291035
Corymbia pocillum	RMF550	MELUD127953a	RMF550	-17.73	141.05	SRR35291030
Corymbia polycarpa	MJB2600	MELUD142136a	MJB2600	-16.31	133.74	SRR35291083

Corymbia polycarpa	MJB2680	MELUD142137a	MJB2680	-16.21	128.37	SRR35291082
Corymbia polycarpa	RMF518	MELUD127921a	RMF518	-18.15	144.63	SRS17943923
Corymbia porrecta	MJB2549a	MELUD114345a	MJB2549a	-13.20	130.71	SRR35291110
Corymbia ptychocarpa subsp. aptycha	CANB5207031	CANB 520703.1	Nicolle, D. 2476	-12.39	133.02	SRR35291062
Corymbia ptychocarpa subsp. aptycha	CANB8173371	CANB 817337.1	Westaway, J.O. 4341	-12.18	136.78	SRR35291031
Corymbia ptychocarpa subsp. ptychocarpa	CANB436269	CANB 436269.1	Hill, K. 4125	-14.70	134.42	SRR35291174
Corymbia ptychocarpa subsp. ptychocarpa	CANB5620451	CANB 562045.1	Nicolle, D. 4248	-16.08	125.76	SRR35291105
Corymbia rhodops	DN1306	AD 99624219	DN1306	-17.38	145.34	SRR35291148
Corymbia serendipita	RMF536	MELUD127939a	RMF536	-18.62	143.82	SRR35291036
Corymbia setosa	D0286337	DNA D0286337	Jobson, P.C. 3294	-17.67	133.62	SRR35291162
Corymbia setosa	QRS128228	QRS 128228.1	Gray, B. 8953	-14.72	143.68	SRR35291195
Corymbia sphaerica	CANB557684	CANB 557684.1	Connors, J.R.; Lepschi, B.J. 1191	-20.62	134.22	SRR35291052
Corymbia stockeri subsp. peninsularis	CNS1361931	CNS 136193.1	Forster, P.I. 34601	-13.88	143.03	SRR35291168
Corymbia stockeri subsp. stockeri	RMF564	MELUD127967a	RMF564	-17.36	144.74	SRR35291029
Corymbia terminalis	MJB2478	MELUD114284a	MJB2478	-23.64	132.87	SRR35291089
Corymbia terminalis	MJB2488	MELUD114287a	MJB2488	-22.08	133.43	SRR35291088
Corymbia terminalis	PD-E40	MELUD127783a	PD-E40	-18.75	125.96	SRR35291070
Corymbia terminalis	RMF528	MELUD127931a	RMF528	-18.73	144.31	SRR35291037
Corymbia trachyphloia	DN2084	AD 163322	DN2084	-32.26	150.43	SRR35291146
Corymbia trachyphloia	RMF507	MELUD127910a	RMF507	-17.40	145.22	SRR35291182
Corymbia umbonata	CANB8173401	CANB 817340.1	Westaway, J. 4413	-13.58	132.49	SRR35291020
Corymbia umbonata	MJB2507	MELUD114306a	MJB2507	-16.58	133.36	SRR35291126
Corymbia xanthope	MEL0293186	MEL 0293186A	Thompson, S.L.; Fox, I.D. 1263	-22.87	149.85	SRR35291050
Corymbia zygophylla	CANB636278	CANB 636278.1	Slee, A.V. 4440	-20.60	118.42	SRR35291132
Corymbia zygophylla	MELU107756	MELUD107756a	Ladiges, P.Y. s.n.	-18.10	124.00	SRR35291095
Eucalyptus behriana	PSF37A	MELUD122586a	PSF37A	-34.46	138.96	SRR35291066
Eucalyptus bigalerita	MJB2156	MELUD114313a	MJB2156	-15.71	133.34	SRR35291090
Eucalyptus brachyandra	MJB2526	MELUD114323a	MJB2526	-15.61	131.09	SRR35291122
Eucalyptus buprestium	SRJ01	MELUD117340a	SRJ01	-34.35	118.15	SRS17943935
Eucalyptus cajuputea	PSF44A	Unvouchered (same population as PSF45N, MELUD122607a)	PSF44A	-33.63	136.60	SRR35291065
Eucalyptus camaldulensis	UoM	MELUD142138a	UoM, RMF s.n.	?	?	SRR35291177
Eucalyptus cladocalyx	MELUD108383	MELUD108383a	Ades, P.; Tibbits, J.; Bayly, M.J. s.n.	?	?	SRR35291091
Eucalyptus cloeziana	129294	QRS 129294.1	Connors, J.R. 1383	-17.39	145.34	SRS17943841
Eucalyptus cloeziana	CNS1418712	CNS 141871.2	Worboys, S.J. 1107	-15.55	144.84	SRS17943859
Eucalyptus cloeziana	MBA3888	MBA 8888.1	Fox, I.D. 2091	-16.23	144.79	SRS17943865
Eucalyptus curtisii	MELUD108358	MELUD108358a	Spokevicius, A.;	?	?	SRS17943908

			Merchant, A. s.n.			
Eucalyptus diversicolor	MELUD108373	MELUD108373a	Spokevicius, A.; Najera, J. s.n.	?	?	SRR35291096
Eucalyptus dumosa	PSF34A	MELUD122581a	PSF34A	-36.13	140.79	SRR35291067
Eucalyptus goniocalyx	MJB2719	MELU	MJB2719	-37.72	145.16	SRR35291081
Eucalyptus guilfoylei	MELUD108359	MELUD108359a	Spokevicius, A.; Najera, J. s.n.	?	?	SRS17943909
Eucalyptus incrassata	SRE05	MELUD117324a	SRE05	-34.30	118.05	SRR35291024
Eucalyptus lehmannii	SRM04	MELUD117362a	SRM04	-34.37	117.79	SRS17943936
Eucalyptus leucophloia	MJB2495	MELUD114293a	MJB2495	-20.19	134.22	SRR35291085
Eucalyptus lirata	MELUD105430a	MELUD105430a	Gibbs, K. s.n.	?	?	SRS17943894
Eucalyptus marginata	SRA02	MELUD117297a	SRA02	-34.39	118.06	SRS17943934
Eucalyptus microcorys	MELUD108375	MELUD108375a	Spokevicius, A .; Tibbits, J .; Bayly, M.J. s.n.	-37.42	143.88	SRS17943911
Eucalyptus miniata	MELUD114324a	MELUD114324a	MJB2527	-15.61	131.12	SRS17943910
Eucalyptus obliqua	MELUD115717	MELUD115717a	LIC03	-38.11	146.44	SRS17943912
Eucalyptus pachyphylla	MJB2490	MELUD114288a	MJB2490	-21.37	133.99	SRR35291087
Eucalyptus raveretiana	DN1297	AD 99624217	DN1297	-19.82	146.79	SRR35291150
Eucalyptus rubiginosa	DN2114	AD 163278	DN2114	-25.19	149.97	SRR35291144
Eucalyptus saligna	MELUD108376	MELUD108376a	Spokevicius, A.; Najera, J. s.n.	?	?	SRR35291092
Eucalyptus socialis	BB511	MELUD127731a	BB511	-24.50	118.58	SRS17943842
Eucalyptus talyuberlup	SRC01	MELUD117309a	SRC01	-34.34	118.11	SRR35291025
Eucalyptus tenuipes	MELUD105466	MELUD105466a	Gibbs, K. s.n.	?	?	SRS17943906
Eucalyptus tetrodonta	RMF575	MELUD127978a	RMF575	-13.89	143.18	SRS17943932
Eucalyptus viminalis	MJB2720	MELUD142141a	MJB2720	-37.71	145.15	SRR35291080

Table S2 Details for samples representing non-monophyletic species, species of anomalous placement, or novel relationships. Sample accession numbers followed by an * indicate herbarium samples that have been re-checked by the authors and verified as correctly identified.

Species	Sample accessions	Investigation and potential explanations for non-monophyly or novel placement
Blakella aspera	MJB2582*, MJB2503*, MBA3091*	Three samples of *B. aspera* are included in the present study; MJB2582 from near Mount Isa, Queensland; MJB2503 from north of Tennant creek, Northern Territory; and MBA3091 from Bowthorn Station, north of Nicholson river, Queensland. Samples MJB2582 and MJB2503 are placed in their own distinct lineage despite ~800 km of geographic separation. However, sample MBA3091 is placed in a separate lineage with samples of *B. polysciada*, *B. candida*, *B. ferriticola* and *B. blakei*. Prior taxonomic works have variously recognised close relationships between *B. aspera* and *B. candida*, *B. ferriticola* and *B. blakei* (Hill and Johnson, 1995; Brooker 2000; Slee et al. 2020; Nicolle 2022). To better understand genetic variation within *B. aspera* and its relationship to other (sister) species, deeper population level sampling from across its range and inclusion of *B. polysciada*, *B. candida*, *B. ferriticola* and *B. blakei* is recommended.
Blakella blakei	MEL0241408A, QRS129092	*Blakella blakei* was previously recognised as two geographic subspecies based on juvenile leaf characters (Hill and Johnson 1995). EUCLID (2020), Nicolle (2022) and Crisp et al. (2024) all recognise a single entity, *B. blakei*, that comprises both subspecies (*B. blakei* subsp. *blakei*, *B. blakei* subsp. *rasilis*) and previously recognised *B. inobvia*. Sampling in the present study represents both former subspecies, *B. blakei* subsp. *rasilis* (MEL0241408A) and *B. blakei* subsp. *blakei* (QRS129092). It is possible that each of these subspecies represents a distinct, non-sister species, resulting in the placement of each sample in very separate lineages in the present study. Further population level sampling of both former subspecies and *B. inobvia*, alongside broader sampling across both lineages containing *B. blakei* is needed to clarify species boundaries.
Blakella polysciada	MEL1590057 MEL1601005A MJB2544*	Two samples of *B. polysciada* (MEL1590057 and MEL1601005) are placed together in a lineage with *B. aspera*, *B. candida*, *B. blakei* and *B. ferriticola*. A third sample of *B. polysciada* (MJB2544) is placed in a separate lineage containing *B. disjuncta* and *B. confertiflora*. Morphologically specimen MJB2544 fits within the description of *B. polysciada*, however the presence of some hairs on specimen crown leaves (mainly along veins) suggests that this sample may represent an intermediate between *B. polysciada* and *B. confertifolia*/*B. disjuncta*, both of which overlap in distribution where this sample was collected.
Blakella tessellaris	RMF497*, RMF509*, CNS1418921*	Two well supported lineages containing *B. tessellaris* samples are evident in the present study. One lineage includes sample RMF497, from the Sunshine Coast, and RMF509 from near Herberton. The other lineage includes a single sample of *B. tessellaris* (CNS1418921) from north of Laura on Cape York Peninsula, placed with two samples of *B. papuana*, both of which were also collected from north of Laura. All three *B. tessellaris* samples have had species identification verified. It is possible that the *B. papauna* samples in this

		study are also *B. tessellaris*, or intermediates between the two species. Eda Addicott notes that *B. papuana* from around this area 'don't look anything like the *papuana* found elsewhere on the Cape York Peninsula'. Regardless of identification of *B. papuana* samples, two distinct and well supported lineages of *B. tessellaris* remain, highlighting the need for thorough population level sampling of *B. tessellaris* (and *B. papuana*) from right across its broad distribution.
Corymbia cadophora	PD_E36<br>CANB640893.1<br>CANB697888.1	Three samples representing all three subspecies of *C. cadophora* were included in the present study. All three samples were placed in a lineage including other species from Hill and Johnson's (1995) series Ferrugineae, and Brooker's (2000) series *Terminalipterae* (*C. abbreviata*, *C. setosa*, *C. deserticola*, *C. zygophylla*, *C. dunlopiana*, *C. pachycarpa*, *C. ferruginea*, *C. sphaerica*, *C. chartaceae*, *C. papillosa*). The western distributed *C. cadophora* subsp. *cadophora* was placed sister to *C. abbreviata*, while the more eastern (and geographically restricted) subspecies' *C. cadophora subsp. pliantha* and *C. cadophora subsp. polychroma* were found to be sister, and most closely related to *C. arenaria*, *C. zygophylla*, *C. chartacea* and *C. papillosa*. The lack of monophyly between *C. cadophora* subsp. *cadophora* and *C. cadophora* subsp. *pliantha* + *polychroma* indicates that taxonomic revision may be required, and further population level sampling is recommended for this species.
Corymbia dichromophloia	PD-E7<br>MJB2528<br>D0286494*	Three samples of *C. dichromophloia* are placed in two separate lineages in this study. Placement of sample D0286494 with *C. umbonata* prompted verification of specimen identification. Fruit size was found to be on the cusp of both *C. umbonata* and *C. dichromophloia*, and without further information on bark character, it was not possible to confirm the sample as either species.
Corymbia. jacobsiana	MEL1610445A<br>MJB2543A	Within *Corymbia*, the placement of *C. jacobsiana* as sister to the remainder of the genus is novel. Morphologically, *C. jacobsiana* is considered unique due to its fibrous, stringy bark (where other *Corymbia* have smooth or tessellated bark). Historically, the species has been placed in its own monospecific section (Hill & Johnson, 1995) or series (Brooker, 2000), but has not been included in any nuclear-based molecular phylogenetic work (i.e. Steane et al., 2002; Ochieng et al., 2007; Parra-O et al., 2009; Schuster et al., 2018; Crisp et al., 2024), apart from Thornhill et al. (2019) where nDNA data was concatenated with ptDNA.
Corymbia stockeri	RMF564,<br>CNS1361931	The two samples of *C. stockeri* included in this study represent each of the two subspecies (*C. stockeri* subsp. *stockeri* and *C. stockeri* subsp. *peninsularis*). Placement of sample CNS1361931 (*C. stockeri* subsp. *peninsularis*) with *C. clarksoniana*, *C. polycarpa*, *C. hylandii*, *C. novoguinensis*, aligns with existing taxonomic concepts (Brooker 2000, Nicolle 2022), noting that *C. stockeri* subsp. *peninsularis* was previously recognised as *C. hylandii* subsp. *peninsularis* (Hill and Johnson 1995). The separate placement of sample RMF564 with two samples of *C. ellipsoidea* is not reflected in current taxonomy. Further molecular study is recommended to confirm the placement of each of these taxa relative to each other and other *Corymbia* species.

Corymbia xanthope	MEL0293186*	In the present study, the single sample of *C. xanthope* is placed in a well supported lineage with *C. erythrophloia*, a species not expected to be sister to *C. xanthope* based on prior taxonomic classification (Hill and Johnson 1995, Nicolle 2022, Slee et al. 2020). Specimen ID of MEL0293186 has been confirmed. At the collection site of MEL0293186 (Marlborough, QLD) both *C. xanthope* and *C. erythrophloia* are present. It is plausible that the relationship presented here is the result of previous hybrid introgression between *C. xanthope* and *C. erythrophloia*. Additional population level sampling of *C. xanthope*, *C. erythrophloia* and *C. hendersonii* (the putative morphological sister species to *C. xanthope*) is needed to clarify these relationships.

Table S3 Recorded natural hybrid combinations in *Angophora*, *Blakella* and *Corymbia* from selected references. Species names are listed as per the reference and their status in EUCLID.

Hybrid (H) or Intergrade (I)	First Hybrid Taxon (Reference)	First Hybrid Taxon (EUCLID)	Second Hybrid Taxon (Reference)	Second Hybrid Taxon (EUCLID)	Reference
H	*A. bakeri*	*A. bakeri*	*A. hispida*	*A. hispida*	Griffin et al. 1988
H	*A. bakeri*	*A. bakeri*	*A. floribunda*	*A. floribunda*	Griffin et al. 1988
H	*A. bakeri*	*A. bakeri*	*A. woodsiana*	*A. woodsiana*	Griffin et al. 1988
H	*A. bakeri*	*A. bakeri*	*A. paludosa*	*A. bakeri*	Griffin et al. 1988
H	*A. bakeri*	*A. bakeri*	*A. floribunda*	*A. floribunda*	Griffin et al. 1988
H	*A. costata*	*A. costata*	*A. hispida*	*A. hispida*	Griffin et al. 1988
H	*A. costata*	*A. costata*	*A. floribunda*	*A. floribunda*	Griffin et al. 1988
H	*A. floribunda*	*A. floribunda*	*A. subvelutina*	*A. subvelutina*	Griffin et al. 1988
H	*A. subvelutina*	*A. subvelutina*	*A. woodsiana*	*A. woodsiana*	Griffin et al. 1988
H	*C. abergiana*	*C. abergiana*	*C. lamprophylla*	*C. lamprophylla*	Hill & Johnson 1995
H	*C. aparrerinja*	*C. aparrerinja*	*C. blakei subsp. blakei-rasilis intergrade*	*C. blakei*	Hill & Johnson 1995
H	*C. aparrerinja*	*C. aparrerinja*	*C. candida subsp. dipsodes*	*C. candida*	Hill & Johnson 1995
H	*C. arafurica*	*C. arafurica*	*C. polysciada*	*C. polysciada*	Hill & Johnson 1995
H	*C. bella*	*C. bella*	*C. confertiflora*	*C. confertiflora*	Hill & Johnson 1995
H	*C. bella*	*C. bella*	*C. grandifolia subsp. grandifolia*	*C. grandifolia subsp. grandifolia*	Hill & Johnson 1995
H	*C. blakei subsp. rasilis*	*C. blakei*	*C. tessellaris*	*C. tessellaris*	Hill & Johnson 1995
H	*C. bleeseri*	*C. bleeseri*	*C. nesophila*	*C. nesophila*	Hill & Johnson 1995
H	*C. bleeseri*	*C. bleeseri*	*C. nesophila*	*C. nesophila*	Griffin et al. 1988
H	*C. bloxsomei*	*C. bloxsomei*	*C. variegata*	*C. citriodora*	Hill & Johnson 1995
H	*C. bloxsomei*	*C. bloxsomei*	*C. citriodora*	*C. citriodora*	Griffin et al. 1988
H	*C. bloxsomei*	*C. bloxsomei*	*C. maculata*	*C. maculata*	Griffin et al. 1988
H	*C. brachycarpa*	*C. brachycarpa*	*C. plena*	*C. plena*	Hill & Johnson 1995
H	*C. brachycarpa*	*C. brachycarpa*	*C. setosa subsp. pedicellaris*	*C. setosa*	Hill & Johnson 1995
H	*C. byrnesii*	*C. greeniana*	*C. nesophila*	*C. nesophila*	Hill & Johnson 1995

H	*C. cadophora*	*C. cadophora*	*C. polycarpa*	*C. polycarpa*	Hill & Johnson 1995
H	*C. calophylla*	*C. calophylla*	*C. chlorolampra*	*C. haematoxylon*	Hill & Johnson 1995
H	*C. calophylla*	*C. calophylla*	*C. ficifolia*	*C. ficifolia*	Hill & Johnson 1995
H	*C. calophylla*	*C. calophylla*	*C. ficifolia*	*C. ficifolia*	Griffin et al. 1988
H	*C. calophylla*	*C. calophylla*	*C. haematoxylon*	*C. haematoxylon*	Griffin et al. 1988
H	*C. candida subsp. dipsodes*	*C. candida*	*C. ferriticola subsp. ferriticola*	*C. ferriticola*	Hill & Johnson 1995
H	*C. candida subsp. lautifolia*	*C. candida x C. flavescens*	*C. flavescens*	*C. flavescens*	Hill & Johnson 1995
H	*C. capricornia*	*C. dichromophloia*	*C. opaca*	*C. terminalis*	Hill & Johnson 1995
H	*C. capricornia*	*C. dichromophloia*	*C. polycarpa*	*C. polycarpa*	Hill & Johnson 1995
H	*C. catenaria*	*C. watsoniana subsp. capillata*	*C. citriodora*	*C. citriodora*	Hill & Johnson 1995
H	*C. chillagoensis*	*C. confertiflora*	*C. confertiflora*	*C. confertiflora*	Hill & Johnson 1995
H	*C. chippendalei*	*C. chippendalei*	*C. eremaea subsp. eremaea*	*C. eremaea*	Hill & Johnson 1995
H	*C. chippendalei*	*C. chippendalei*	*C. opaca*	*C. terminalis*	Hill & Johnson 1995
H	*C. citriodora*	*C. citriodora*	*C. watsoniana*	*C. watsoniana*	Griffin et al. 1988
H	*C. citriodora*	*C. citriodora*	*C. maculata*	*C. maculata*	Griffin et al. 1988
H	*C. clarksoniana*	*C. clarksoniana*	*C. hylandii subsp. hylandii*	*C. hylandii*	Hill & Johnson 1995
H	*C. clavigera*	*C. clavigera*	*C. grandiflora*	?	Griffin et al. 1988
H	*C. collina*	*C. collina*	*C. perfoliata*	*C. cadophora subsp. cadophora*	Griffin et al. 1988
H	*C. confertiflora*	*C. confertiflora*	*C. papuana*	*C. papuana*	Griffin et al. 1988
H	*C. confertiflora*	*C. confertiflora*	*C. tessellaris*	*C. tessellaris*	Hill & Johnson 1995
H	*C. curtipes*	*C. greeniana*	*C. opaca*	*C. terminalis*	Hill & Johnson 1995
H	*C. curtipes*	*C. greeniana*	*C. polycarpa*	*C. polycarpa*	Hill & Johnson 1995
H	*C. curtipes*	*C. greeniana*	*C. terminalis*	*C. terminalis*	Hill & Johnson 1995
H	*C. curtipes*	*C. greeniana*	*C. umbonata*	*C. umbonata*	Hill & Johnson 1995
H	*C. dallachiana*	*C. dallachiana*	*C. dimorpha*	*C. peltata*	Hill & Johnson 1995
H	*C. dallachiana*	*C. dallachiana*	*C. gilbertensis*	*C. gilbertensis*	Hill & Johnson 1995
H	*C. dampieri*	*C. greeniana*	*C. polycarpa*	*C. polycarpa*	Hill & Johnson 1995
H	*C. dendromerinx*	*C. dendromerinx*	*C. flavescens*	*C. flavescens*	Hill & Johnson 1995

H	*C. deserticola subsp. mesogeotica*	*C. deserticola subsp. mesogeotica*	*C. eremaea subsp. oligocarpa*	*C. eremaea*	Hill & Johnson 1995
H	*C. dichromophloia*	*C. dichromophloia*	*C. latifolia*	*C. latifolia*	Hill & Johnson 1995
H	*C. dichromophloia*	*C. dichromophloia*	*C. foelscheana*	*C. foelscheana*	Griffin et al. 1988
H	*C. dichromophloia*	*C. dichromophloia*	*C. terminalis*	*C. terminalis*	Griffin et al. 1988
H	*C. dimorpha*	*C. peltata*	*C. leichhardtii*	*C. leichhardtii*	Hill & Johnson 1995
H	*C. dimorpha*	*C. peltata*	*C. peltata*	*C. peltata*	Hill & Johnson 1995
H	*C. disjuncta*	*C. disjuncta*	*C. papuana*	*C. papuana*	Hill & Johnson 1995
H	*C. disjuncta*	*C. disjuncta*	*C. polysciada*	*C. polysciada*	Hill & Johnson 1995
H	*C. dolichocarpa*	*C. clarksoniana*	*C. tumescens*	*C. dichromophloia*	Hill & Johnson 1995
H	*C. drysdalensis*	*C. dichromophloia*	*C. greeniana*	*C. greeniana*	Hill & Johnson 1995
H	*C. drysdalensis*	*C. dichromophloia*	*C. polycarpa*	*C. polycarpa*	Hill & Johnson 1995
H	*C. foelscheana*	*C. foelscheana*	*C. polycarpa*	*C. polycarpa*	Hill & Johnson 1995
H	*C. foelscheana*	*C. foelscheana*	*C. polycarpa*	*C. polycarpa*	Griffin et al. 1988
H	*C. grandifolia*	*C. grandifolia*	*C. papuana*	*C. papuana*	Griffin et al. 1988
H	*C. greeniana*	*C. greeniana*	*C. polycarpa*	*C. polycarpa*	Hill & Johnson 1995
H	*C. gummifera*	*C. gummifera*	*C. maculata*	*C. maculata*	Hill & Johnson 1995
H	*C. gummifera*	*C. gummifera*	*C. maculata*	*C. maculata*	Griffin et al. 1988
H	*C. henryi*	*C. henryi*	*C. torelliana*	*C. torelliana*	Hill & Johnson 1995
H	*C. henryi*	*C. henryi*	*C. variegata*	*C. citriodora*	Hill & Johnson 1995
H	*C. intermedia*	*C. intermedia*	*C. maculata*	*C. maculata*	Hill & Johnson 1995
H	*C. latifolia*	*C. latifolia*	*C. novoguinensis*	*C. novoguinensis*	Hill & Johnson 1995
H	*C. latifolia*	*C. latifolia*	*C. oocarpa*	*C. oocarpa*	Hill & Johnson 1995
H	*C. maculata*	*C. maculata*	*C. watsoniana*	*C. watsoniana*	Griffin et al. 1988
H	*C. paracolpica*	*C. papuana*	*C. tessellaris*	*C. tessellaris*	Hill & Johnson 1995
H	*C. peltata subsp. leichhardti*	*C. peltata*	*C. watsoniana*	*C. watsoniana*	Griffin et al. 1988
H	*C. plena*	*C. plena*	*C. terminalis*	*C. terminalis*	Hill & Johnson 1995
H	*C. pocillum*	*C. pocillum*	*C. polycarpa*	*C. polycarpa*	Hill & Johnson 1995
H	*C. polycarpa*	*C. polycarpa*	*C. ptychocarpa subsp. aptycha*	*C. ptychocarpa subsp. aptycha*	Hill & Johnson 1995

H	*C. polycarpa*	*C. polycarpa*	*C. terminalis*	*C. terminalis*	Hill & Johnson 1995
H	*C. polycarpa*	*C. polycarpa*	*C. setosa*	*C. setosa*	Griffin et al. 1988
H	*C. polycarpa*	*C. polycarpa*	*C. ptychocarpa*	*C. ptychocarpa*	Griffin et al. 1988
H	*C. tessellaris*	*C. tessellaris*	*C. torelliana*	*C. torelliana*	Hill & Johnson 1995
H	*C. variegata*	*C. citriodora*	*C. watsoniana subsp. watsoniana*	*C. watsoniana subsp. watsoniana*	Hill & Johnson 1995
I	*C. aparerrinja*	*C. aparrerinja*	*C. dallachiana*	*C. dallachiana*	Hill & Johnson 1995
I	*C. aparrerinja*	*C. aparrerinja*	*C. blakei subsp. blakei subsp. rasilis*	*C. blakei*	Hill & Johnson 1995
I	*C. aparrerinja*	*C. aparrerinja*	*C. flavescens*	*C. flavescens*	Hill & Johnson 1995
I	*C. arafurica*	*C. arafurica*	*C. bella*	*C. bella*	Hill & Johnson 1995
I	*C. bella*	*C. bella*	*C. paracolpica*	*C. papuana*	Hill & Johnson 1995
I	*C. byrnesii*	*C. greeniana*	*C. greeniana*	*C. greeniana*	Hill & Johnson 1995
I	*C. capricornia*	*C. dichromophloia*	*C. drysdalensis*	*C. dichromophloia*	Hill & Johnson 1995
I	*C. capricornia*	*C. dichromophloia*	*C. umbonata*	*C. umbonata*	Hill & Johnson 1995
I	*C. catenaria*	*C. watsoniana subsp. capillata*	*C. watsoniana subsp. capillata*	*C. watsoniana subsp. capillata*	Hill & Johnson 1995
I	*C. citriodora*	*C. citriodora*	*C. variegata*	*C. citriodora*	Hill & Johnson 1995
I	*C. clarksoniana*	*C. clarksoniana*	*C. novoguinensis*	*C. novoguinensis*	Hill & Johnson 1995
I	*C. confertiflora*	*C. confertiflora*	*C. disjuncta*	*C. disjuncta*	Hill & Johnson 1995
I	*C. curtipes*	*C. greeniana*	*C. foelscheana*	*C. foelscheana*	Hill & Johnson 1995
I	*C. curtipes*	*C. greeniana*	*C. greeniana*	*C. greeniana*	Hill & Johnson 1995
I	*C. dallachiana*	*C. dallachiana*	*C. grandifolia subsp. grandifolia*	*C. grandifolia subsp. grandifolia*	Hill & Johnson 1995
I	*C. dampieri*	*C. greeniana*	*C. greeniana*	*C. greeniana*	Hill & Johnson 1995
I	*C. disjuncta*	*C. disjuncta*	*C. papuana*	*C. papuana*	Hill & Johnson 1995
I	*C. disjuncta*	*C. disjuncta*	*C. pauciseta*	*C. pauciseta*	Hill & Johnson 1995
I	*C. dolichocarpa*	*C. clarksoniana*	*C. maritima*	*C. clarksoniana*	Hill & Johnson 1995
I	*C. dolichocarpa*	*C. clarksoniana*	*C. plena*	*C. plena*	Hill & Johnson 1995
I	*C. drysdalensis*	*C. dichromophloia*	*C. opacula*	*C. terminalis x C. dichromophloia*	Hill & Johnson 1995
I	*C. eremaea subsp. eremaea*	*C. eremaea*	*C. opaca*	*C. terminalis*	Hill & Johnson 1995
I	*C. eremaea subsp. oligocarpa*	*C. eremaea*	*C. opaca*	*C. terminalis*	Hill & Johnson 1995

I	*C. erythrophloia*	*C. erythrophloia*	*C. pocillum*	*C. pocillum*	Hill & Johnson 1995
I	*C. flavescens*	*C. flavescens*	*C. grandifolia subsp. grandifolia*	*C. grandifolia subsp. grandifolia*	Hill & Johnson 1995
I	*C. hamersleyana*	*C. hamersleyana*	*C. semiclara*	*C. hamersleyana*	Hill & Johnson 1995
I	*C. lenziana*	*C. lenziana*	*C. opaca*	*C. terminalis*	Hill & Johnson 1995
I	*C. maculata*	*C. maculata*	*C. variegata*	*C. citriodora*	Hill & Johnson 1995
I	*C. novoguinensis*	*C. novoguinensis*	*C. polycarpa*	*C. polycarpa*	Hill & Johnson 1995
I	*C. opaca*	*C. terminalis*	*C. terminalis*	*C. terminalis*	Hill & Johnson 1995
I	*C. terminalis*	*C. terminalis*	*C. tumescens*	*C. terminalis*	Hill & Johnson 1995
